# Transcriptomic analyses of patient peripheral blood with hemoglobin depletion reveal glioblastoma biomarkers

**DOI:** 10.1038/s41525-022-00348-3

**Published:** 2023-01-25

**Authors:** Dan Qi, Yiqun Geng, Jacob Cardenas, Jinghua Gu, S. Stephen Yi, Jason H. Huang, Ekokobe Fonkem, Erxi Wu

**Affiliations:** 1 Department of Neurosurgery and Neuroscience Institute, Baylor Scott & White Health, Temple, TX 76508 USA; 2 Laboratory of Molecular Pathology, Shantou University Medical College, 515041 Shantou, China; 3 Baylor Scott & White Research Institute, Dallas, TX 75204 USA; 4 Institute for Cellular and Molecular Biology (ICMB), College of Natural Sciences, The University of Texas at Austin, Austin, TX 78712 USA; 5 Oden Institute for Computational Engineering and Sciences (ICES), The University of Texas at Austin, Austin, TX 78712 USA; 6 Department of Biomedical Engineering, Cockrell School of Engineering, The University of Texas at Austin, Austin, TX 78712 USA; 7 Department of Oncology, LIVESTRONG Cancer Institutes, Dell Medical School, The University of Texas at Austin, Austin, TX 78712 USA; 8 Texas A & M University School of Medicine, Temple, TX 76508 USA; 9 Texas A & M University School of Pharmacy, College Station, TX 77843 USA

**Keywords:** Diagnostic markers, CNS cancer

## Abstract

Peripheral blood is gaining prominence as a noninvasive alternative to tissue biopsy to develop biomarkers for glioblastoma (GBM); however, widely utilized blood-based biomarkers in clinical settings have not yet been identified due to the lack of a robust detection approach. Here, we describe the application of globin reduction in RNA sequencing of whole blood (i.e., WBGR) and perform transcriptomic analysis to identify GBM-associated transcriptomic changes. By using WBGR, we improved the detection sensitivity of informatic reads and identified differential gene expression in GBM blood. By analyzing tumor tissues, we identified transcriptomic traits of GBM blood. Further functional enrichment analyses retained the most changed genes in GBM. Subsequent validation elicited a 10-gene panel covering mRNA, long noncoding RNA, and microRNA (i.e., GBM-Dx panel) that has translational potential to aid in the early detection or clinical management of GBM. Here, we report an integrated approach, WBGR, with comprehensive analytic capacity for blood-based marker identification.

## Introduction

Glioblastoma multiforme (GBM) is the most common, aggressive and heterogeneous type of adult primary brain cancer, aka malignant brain glioma (WHO grade IV), which occurs in the human central nervous system (CNS) and has poor survival. Despite multimodal treatments such as surgery followed by radiochemotherapy, the median survival time for patients with GBM is ~15 months^1–5^. Both effective therapeutics and reliable biomarkers are desirable to improve patient survival outcomes for this malignancy.

Liquid biopsy is rapidly emerging as a promising medium for cancer detection, treatment monitoring and prognosis prediction. Blood components such as serum, plasma, circulating tumor cells (CTCs), microvesicles, and cell-free (cf) nucleic acids have been intensively investigated for biomarker development for GBM^6–15^. For instance, studies from Muller et al. and Sullivan et al. revealed detectable CTCs in blood from ~20.6% and ~39.4% of GBM patients, respectively^6,7^. Wang et al. identified two fusion transcripts (*FGFR3-TACC3* and *VTI1A-TCF7L2*) in both GBM tissue and matched plasma samples^16^. Sabedot et al. showed a cfDNA-derived methylation signature that reflected clinicopathological changes during glioma posttreatment surveillance^17^. For brain tumors, cerebrospinal fluid (CSF) is also a promising material to develop biomarkers. An early study reported that the detection of seven microRNAs (miRNAs), i.e., miR-10b, miR-21, miR-125b, miR-141, miR-200a, miR-200b, and miR-200c, in CSF showed over 90% accuracy in differentiating GBM from metastatic brain tumors^18^. Promoter hypermethylation in *MGMT*, *CDKN2A*, *TIMP-3*, and *THBS1* was detected in CSF, serum, and tumor tissue specimens in GBM patients but not in healthy donors^19,20^. These efforts and findings support the idea of identifying liquid biomarkers for GBM patients; however, as few markers derived from blood components have entered the clinical validation phase, continued efforts and different methods are needed.

Peripheral whole blood has also drawn increasing interest in biomarker discovery due to the presence of circulating tumorous cells, tumor-interacting cells and disease-related immune cells, and none of its components are separated or removed^21^. Given that blood can reflect physiological and pathological events occurring in other tissues and organs of the body, unlike serum and plasma, whole blood is considered to be able to deliver a comprehensive view of the status of the immune system^22,23^. However, surprisingly, whole blood is rarely studied for biomarker discovery of GBM. We reasoned that whole blood contains a high level of hemoglobin, making up a high background for analyses, such as transcriptome profiling, and as a result, a robust detection method is lacking. Indeed, our previous work and a later study from another group showed that hemoglobin RNAs account for more than half (a majority) of the total transcripts from a whole blood sample^24,25^. Additionally, brain tumors are protected by the blood-brain barrier (BBB), although they may not be intact. Therefore, circulating biomarkers in liquid biopsy are significantly lower than those in nonbrain lesions^26^. Thus, we consider that globin reduction (GR) is an innovative idea to conduct whole blood biomarker studies to reduce noise and enrich GBM-associated factors. Our previous work showed that GR prior to high-throughput differential gene expression (DGE) analysis was able to dramatically improve the sensitivity of sex marker detection in human whole blood^24^. Therefore, in this study, we explored whether hemoglobin depletion prior to transcriptome profiling of blood RNA improves the detection sensitivity of GBM-associated transcriptomic changes.

**Fig. 1:**
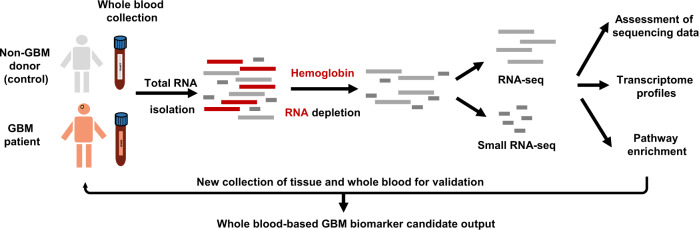
Schematic overview of the WBGR workflow. We performed globin reduction on RNA isolated from 22 individuals and RNA-seq on 28 RNA samples derived from them. We assessed the sequencing data from the pre-GR group and post-GR group and performed differential transcriptomic analysis on all post-GR samples. We then conducted functional enrichment analysis and identified potential GBM-associated transcriptomic traits.

To this end, we first performed hemoglobin RNA depletion prior to transcriptome sequencing for RNAs isolated from whole blood and assessed the merits of GR in blood RNA-seq (Fig. 1). We then conducted a comprehensive transcriptomics analysis for post-GR GBM blood, a blood-tumor tissue merging analysis of signaling pathways and perturbations reflected in the blood, a modular enrichment analysis of immune-related gene expression changes and validation analyses of candidate GBM biomarkers at both the testing cohort level and the individual patient level.

## Results

### Advantages of globin reduction (GR) in blood RNA-seq

**Fig. 2:**
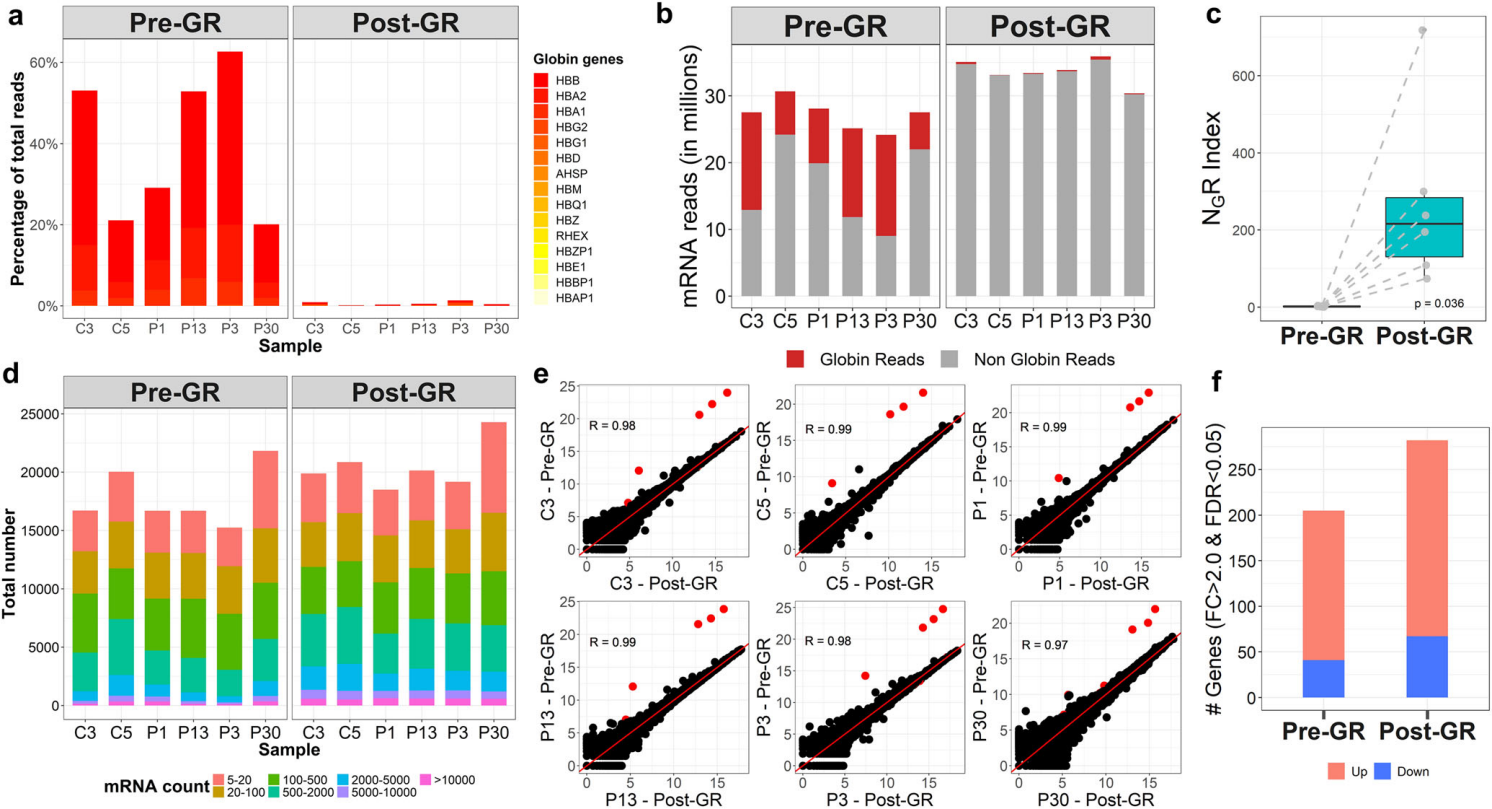
Globin reduction increases sequencing sensitivity. **a** Stacked bars showing globin gene reads in pre- or post-GR samples. Hemoglobin genes are shown in the legends beside the plots. **b** Bar plots showing the number of mRNA reads for samples before and after GR. The red segments represent reads from globin genes. **c** Boxplots showing the N_G_RI scores in the pre- and post-GR groups. Box limits indicate the range of the central 50% of the data with the central line marking the median value. Light gray dots show the N_G_RI scores in each group. Dashed lines connect the N_G_RI scores for pre- and post-GR samples from the same donor. A paired *t*-test was used for comparison. **d** Stacked bars showing total counts of aligned genes in pre- and post-GR samples. Count categories are genes with 5–20 counts, 20–100 counts, 100–500 counts, 500–2000 counts, 2000–5000 counts, 5000–10000 counts, or >10000 counts per sample. **e** Sample-level scatterplots comparing log2 normalized counts before and after GR, with post-GR samples plotted along the *x*-axis and pre-GR samples plotted along the *y*-axis. The red lines have slope = 1 and pass through the origin, and the red dots represent globin genes. Pearson’s correlation coefficient (R) for each scatterplot is shown. **f** Bar plots showing the number of DEGs surviving FDR ≤ 0.05 before and after GR. The red segments represent upregulated genes, while blue represents downregulated genes.

Hemoglobin is the most abundant protein in blood, potentially making up a high background of reads in individual blood samples during probe-based analysis or next-generation sequencing analysis^24^. To identify GBM-associated gene expression features through the comparison between GBM patients and non-GBM individuals by RNA-seq, we first assessed the impact of GR on RNA-seq results. Peripheral blood RNA samples from six donors, each with and without GR, were analyzed. Total RNA was isolated from donor blood. Subsequent hemoglobin mRNA depletion was performed, and the resulting samples were then subjected to RNA-seq. Briefly, half of the blood RNA from each donor was left as a pre-GR control, while the remaining half of the blood RNA was subjected to GR. The resultant RNA-seq reads were used to analyze whether GR prompts more informative RNA-seq results. The decrease in globin mRNA reads was first analyzed and confirmed in a comparison between pre- and post-GR samples (Fig. 2a). As shown in Fig. 2b, GR reduced the percentage of total reads dominated by hemoglobin genes from an average of 39.8% (ranging from 20.0% to 62.6%) per sample to an average of less than 1% per sample. This reveals that hemoglobin mRNA reads can make up to 63% in a single blood RNA sample and can be largely different in patients. A similar observation was reported in a previous study that showed that hemoglobin transcripts ranged from 52% to 76% in their investigated subject cohort^25^. To better evaluate the benefit of reducing globin noise on meaningful read counts, we computed a globin noise reduction index (N_G_RI) for each sample and found a significant increase in N_G_RI values from an average of ~2.1 per sample in the pre-GR group to an average of ~272.0 per sample in the post-GR group (Fig. 2c). Count-level analysis confirmed a read increase in all count categories, not only low abundance genes, as indicated in Fig. 2d.

The gene expression levels pre-GR and post-GR in a given sample were then analyzed by Pearson correlation and visualized using scatter plots (Fig. 2e). The high correlation coefficient of concordance of at least R ≥ 0.97 indicates that GR did not introduce bias to the expression detection of non-globin genes. Comparing GBM samples and control samples in the pre-GR group, no hemoglobin gene survived after differential gene expression (DGE) analysis, indicating the importance of GR in blood differential transcriptome analysis. Meanwhile, GR increased the number of non-globin mRNA reads by an average of over twofold for both upregulated and downregulated genes (Fig. 2f). DGE analysis identified ~31.5% more GBM-related differentially expressed genes (DEGs) (FDR ≤ 0.05) in post-GR samples than in pre-GR samples (Fig. 2f). Together, our results reveal that without GR, hemoglobin reads account for up to 63% of total mRNA reads and demonstrate that GR decreases the inefficient analysis background while increasing the detection sensitivity and improving the informative reads from blood RNA-seq.

### DGE analysis of RNA-seq data for post-GR GBM blood

**Fig. 3:**
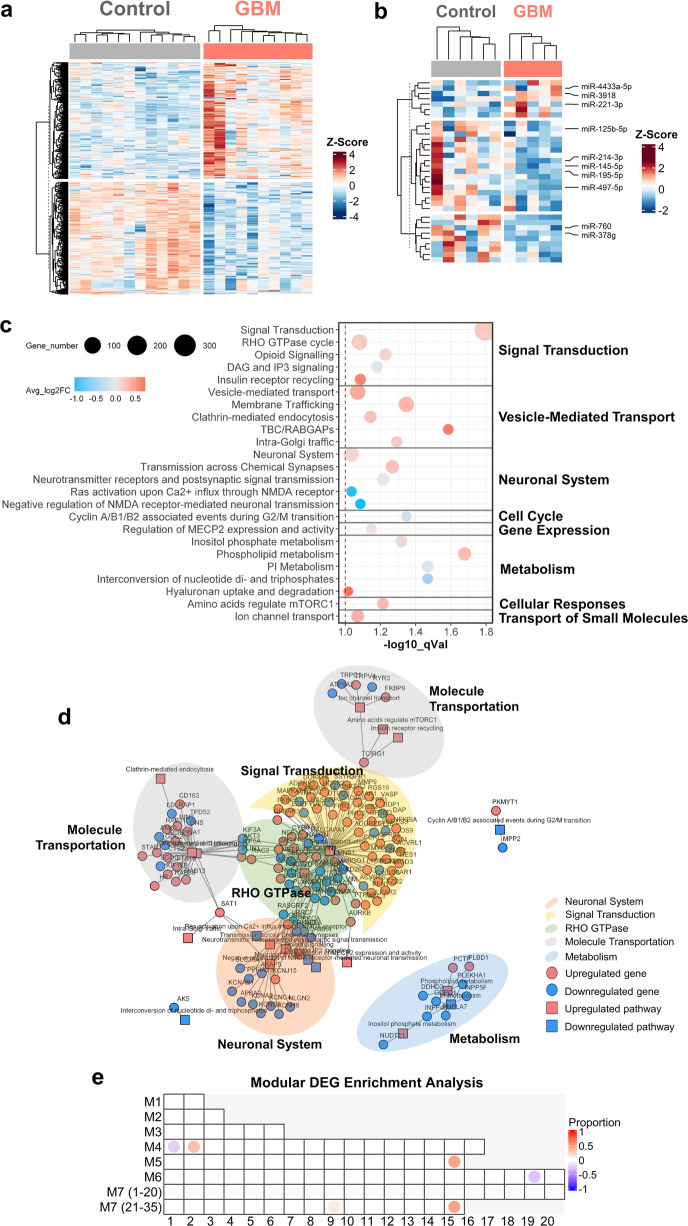
Gene expression profiling and integrated functional network analyses. **a** Heatmap of differentially expressed mRNAs that survived FDR ≤ 0.05 and FC ≥ 2.0. **b** Heatmap of log2 normalized counts for miRNAs that survived *P* ≤ 0.05 and FC ≥ 2.5. For each gene in **a** or **b**, the mean of the control group was subtracted. Hierarchical clustering was performed for the heatmaps. **c** Dot plot showing the functional annotation of overlapping Reactome signaling pathways in both GBM blood RNA-seq results and GBM tissue datasets. The *x*-axis is the −log10 q-value for each pathway. The red dotted line represents the −log10 transformed *q-*values (FDR) of 0.1. **d** Network connecting DEGs and major overlapping pathways shown in **a**. **e** DEG enrichment analysis using the modular enrichment method. The proportions of DEGs in modules are indicated by a color gradient ranging from blue (100% of transcripts decreased) to red (100% of transcripts increased). C control, P GBM patient.

To analyze the transcriptomic profiling differences between the control and GBM groups, the sequencing data of all post-GR samples were analyzed. The overall analytic workflow and an overview of data processing are shown in Supplementary Figs. 1–3. DGE analysis was performed using RNA-seq data from all post-GR samples. Comparisons were performed with an adjustment for sex. Changes in mRNAs with FDR ≤ 0.05 and microRNAs (miRNAs) with *p-*value ≤0.05 were considered significant, i.e., differentially expressed. DEGs and differentially expressed miRNAs (DEMs) with a fold change (FC) of at least over 2.0 were selected and used for further analysis and data visualization. There were 487 genes (250 upregulated, 237 downregulated) that survived the cutoff of FC ≥ 2.0 and FDR ≤ 0.05 (Fig. 3a), and 33 miRNAs (7 upregulated, 23 downregulated) survived the cutoff of FC ≥ 2.5 and *p-*value *≤* 0.05 (Fig. 3b). Tumor-promoting miRNAs such as miR-221-3p and tumor-suppressing miRNAs such as miR-497-5p and miR-195-5p were among our identified DEMs in GBM blood^27–29^. Next, by retrieving validated targets of the DEMs using multiple miRNA-target databases in the multiMiR R package^30^, 196 blood DEGs were identified as targets of these blood DEMs.

### Merging DGE analysis of GBM blood RNA-seq data and external GBM tissue transcriptome profiling data

To assess the overlap of gene expression in whole blood and tissue samples, we accessed and analyzed the transcriptome profiling data of GBM tumor tissues. The Cancer Genome Atlas (TCGA) database was accessed to download RNA-seq data for 156 primary GBM tissues (TCGA-GBM project) and 5 solid normal tissue controls^31^. DGE analysis comparing GBM tumor tissues with normal tissue controls revealed a total of 9314 DEGs (FC > 2.0 and FDR ≤ 0.05) in TCGA-GBM tumor tissues. Overlapping genes were found in the DEGs identified from GBM tissues and the DEGs identified from GBM blood. Additionally, we queried the R2 Genomics Analysis and Visualization Platform to identify DEGs in R2 GBM tissue datasets. Two normal brain tissue datasets, including a total of 216 non-tumor brain samples, and three GBM tumor tissue datasets, including a total of 200 GBM tumor tissue samples, were compared. A total of 5038 DEGs that survived FC > 2.0 and FDR ≤ 0.05 were identified. We then performed a merging analysis of all blood DEGs identified from RNA-seq with all tissue DEGs identified from TCGA and R2 databases and found 148 shared DEGs in both tissue and blood data. Details of these datasets are provided in Supplementary Table 3 and the Methods section. Blood DEGs surviving at least two of the above analyses were retained. This result indicates that gene expression in whole blood is able to reflect GBM tissue features, which confirms the existence of circulating molecules in GBM blood and strongly supports the possibility of blood biomarker identification for brain tumors such as GBM.

### Functional enrichment analysis of DEGs from GBM blood and tissues

To further characterize the main biological functions and key pathways involving the DEGs identified from GBM blood and external GBM tissue datasets, functional enrichment analysis was performed via Reactome^32^. The enriched signaling pathways were selected with thresholds of FDR < 0.1 and FC > 1.4 (considering that a significant change may not occur for every participant in cellular signaling). A previous study analyzing GBM tumor tissues revealed that genes involved in RTK (receptor tyrosine kinases) signaling, PI3K (phosphoinositide 3-kinase) signaling, MAPK (mitogen-activated protein kinase) signaling, and p53 and RB1 (retinoblastoma gene) signaling pathways were frequently mutated in GBM tumor cells, suggesting that dysfunction of these pathways may be largely associated with GBM^31,33^. Comparison of perturbed signaling pathways between blood and tissue data revealed that several subsignaling pathways involved in signal transduction (e.g., RHO GTPase cycle), neuronal system (e.g., N-methyl-D-aspartate receptor [NMDAR] signaling), cell cycle, transport of small molecules, metabolism, and so on, which have close links with the reported GBM-associated pathways mentioned above, are reflected in GBM blood (Fig. 3c). For example, the RHO GTPase cycle, which can be regulated by RTK signaling, is upregulated, NMDAR signaling can be coupled with ERK/MAPK signaling, and the negative regulation of NMDAR-mediated signaling is downregulated in GBM blood (Fig. 3c). The gene network connecting DEGs involved in these pathways is shown in Fig. 3d. These observations suggest that some common tumor-related alterations of signaling pathways can be detected in blood, supporting the application of whole blood samples for biomarker development for GBMs.

### Baylor Modular Analysis of DEGs functioning in the immune system

From Baylor Modular Analysis, a unique blood gene modular analysis by colleagues at our institute Baylor Scott & White Health, immune-related gene sets that changed in a coordinated manner were clustered into modules to facilitate blood transcriptome analyses^34,35^. To enrich DEGs related to the immune system in GBM blood, we took advantage of these module repertoires and analyzed our identified GBM blood DEGs to identify significantly changed immune-related genes. The blood DEGs identified with FC ≥ 2.0 were subjected to this modular enrichment analysis. As shown in Fig. 3e, the most significant differences (FDR < 0.05) were observed in modules M4.1, M4.2, M5.15, M6.19, M7.29, and M7.35, and the genes in these modules were then selected for further analysis. The module numbers show the selection round of transcripts, and the modules are more specific as the number rises. For example, module M1 is generic and may change in many diseases^34^. Among the modules identified above, M4.1 showed genes that are involved in T cell function, M4.2 showed genes that are involved in inflammation, M5.15 showed genes that are linked to neutrophils, and some modules have no specific annotations but are involved in the immune system or act as interactors. From this analysis, we identified immune-related DEGs in GBM blood. For example, *CCR7* and *NTSR1* were downregulated, and *VNN1*, *C2, C1QB, SLC2A5, HLA-G, KIR2DL4*, and *NDST1* were upregulated. *SLC2A5* was shown by Wei et al. to be expressed at higher levels in GBM-associated macrophages than in monocytes from GBM patients or healthy donors^36^. *HLA-G* was recently found to be highly expressed in some cancer types regulating immune escape from cytotoxic T cells^37,38^. Additionally, our recent analysis of human immunome alterations identified *HLA-G* as a novel immune response gene across cancer types^39^. These studies support our approach in capturing GBM features in blood and emphasize the need for further in-depth functional studies of these genes.

**Fig. 4:**
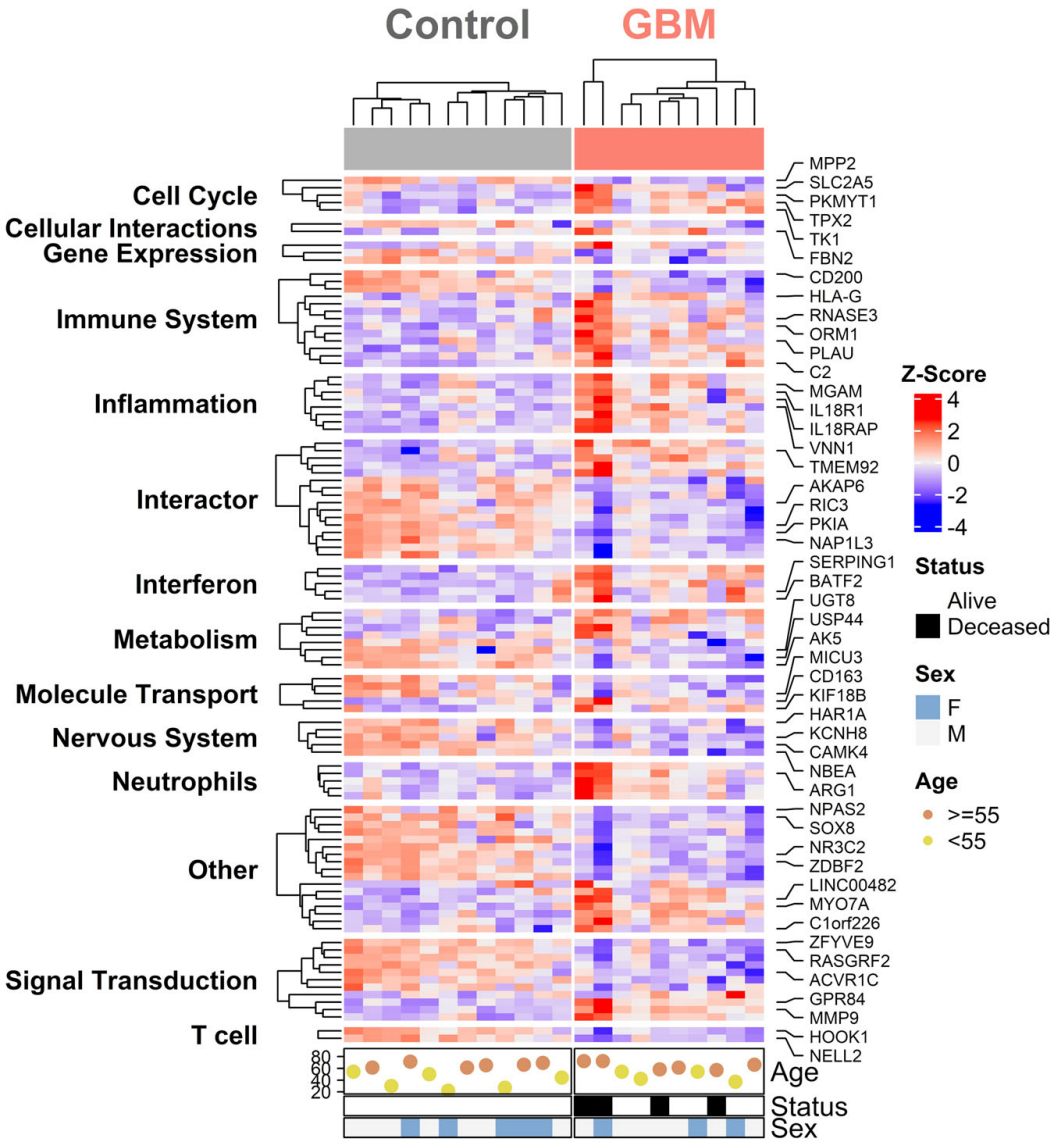
GBM-associated DEGs. Heatmap of 105 narrow-downed DEGs in GBM blood samples from comprehensive analyses. Ages over 55 years are shown as brown dots. Hierarchical clustering was also applied. Genes were separated into blocks according to their potential functions or related pathways. C control, P GBM patient.

Together, after the integrated analysis of DEG characterization, Reactome pathway analysis, and Baylor Modular Analysis, 105 DEGs in GBM blood were further enriched and summarized with patient clinical data, including age, sex, and vital status, in a heatmap shown in Fig. 4. Some genes with annotated biological functions are highlighted alongside the heatmap.

### qPCR validation of a panel of genes in independent whole blood and tissue specimens

To further identify GBM-specific genes, including miRNAs, from the aforementioned analyses, whole blood samples from two healthy individuals and five GBM patients were further collected, and tumor tissue and normal adjacent tissue samples from ten GBM patients were obtained from our hospital’s Brain Bank. These samples were used as independent testing samples for validation.

**Fig. 5:**
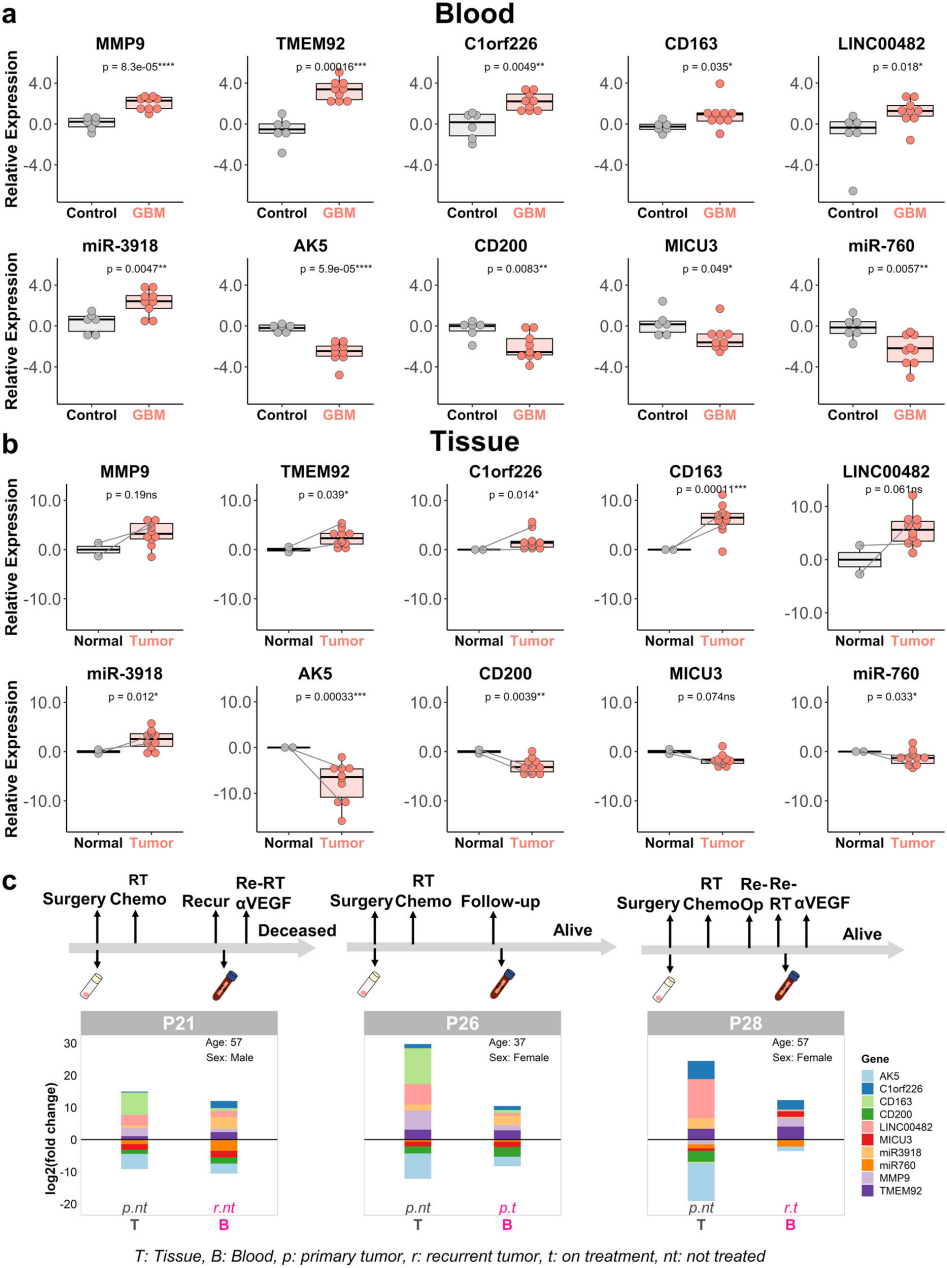
RT-qPCR validation of selected genes and miRNAs in newly collected blood samples and tissue samples. **a**, **b** Mean values of multiple PCR results from blood (**a**) and tissue (**b**) samples are shown in boxplots. Data for controls are shown with dots in gray, and data for GBMs are shown with dots in salmon. Box limits indicate the range of the central 50% of the data (from 75th to 25th quantiles) with the central line marking the median value. Comparisons (one-sided *t*-test): GBM vs. control (**a**) or tumor vs. normal (**b**). Data for samples from the same patient are connected by gray right arrow lines. **c** Expression of the GBM-Dx panel in the blood and tumor tissue of three GBM patients is shown in stacked bar plots. P GBM patient, Age age at blood draw, RT radiation therapy, Chemo chemotherapy, Re-RT reirradiation, Re-Op reoperation or reresection, αVEGF anti-VEGF/anti-angiogenetic therapy.

To enrich a common trait in GBM blood, we selected genes, including mRNAs, lncRNAs, and miRNAs, that showed a higher frequency across all GBM samples (>50%), and at least 50% of the selected genes were set to be upregulated. Such genes were selected to design primers, and primers that target all transcripts of the only given gene were designed and ordered or primers that are reported in other publications were used. We selected eight DEGs and two DEMs to perform RT-qPCR experiments. PCR results were normalized by the mean of the control group and then log2 transformed for visualization. Blood samples that were used in RNA-seq, including four controls (C4, C20, C24, and C38) and four GBMs (P1, P21, P26, and P37), were tested in the qPCR assay again as methodological positive controls. Blood samples C24, C38, and P1, which were not used in miRNA-seq, were considered additional testing samples in miRNA validation. From our results, the gene expression pattern of these samples in qPCR data was consistent with RNA-seq data, which verified the reliability of the qPCR used in this work. The qPCR results are summarized in Fig. 5a. To evaluate the expression of these genes in GBM tumor cells, ten tumor tissues and two normal adjacent tissues from GBM patients were also used to perform qPCR. The results are summarized in Fig. 5b. Data from the same patient are connected by gray right arrow lines in Fig. 5b. qPCR results in triplicate for each sample are provided as bar graphs in Supplementary Fig. 4. In the investigated patient cohort, we had both tumor tissue and blood samples available for three patients (P21, P26, and P28); thus, we performed a patient-level analysis of the ten candidate marker genes. qPCR data for the three patients were plotted as stacked bar charts (Fig. 5c). The data show that the expression of the ten genes from blood samples reflected their expression pattern in their matched tumor tissues, especially prior to treatment. The expression of 10 genes shows a similar scale in the recurrent blood and primary tumor tissue samples of patient P21, whose survival is less than 2 years after being diagnosed with GBM. The other two patients, who showed a reduced overall panel gene expression scale in blood after treatment compared to that in pretreatment tumors, are still alive with a survival of over 3 years. This result demonstrates that GBM-associated gene expression is detectable in patient blood and suggests that such an expression pattern has an indicative value for patient outcome.

**Fig. 6:**
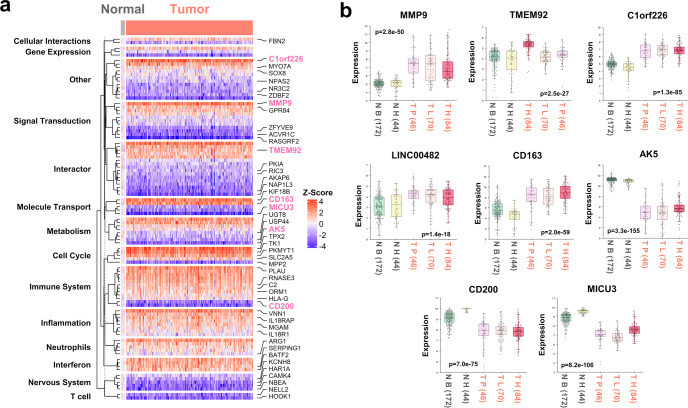
Expression comparison of selected genes using TCGA-GBM transcriptome profiling data and R2 genomics RNA profiling data. **a** Expression of 98 genes from the 105 DEGs shown in main Fig. 4 in TCGA-GBM tissue data (22 genes are not available in TCGA). Potential function or pathway annotations are labeled on the left side of the heatmap. Genes in the GBM-Dx panel are labeled in hot pink. **b** The R2 genomics database was queried for eight genes (in the GBM-Dx panel) comparing normal brain data and GBM tissue data. The GBM patient cohorts used are “N B (172)”, representing the N Brain 172 (Berchtold) dataset, “N H (44)”, representing the N Brain 44 (Harris) dataset as the normal control group, “T P (46)”, representing the T Glioblastoma 46 (Pfister) dataset, “T L (70)”, representing the T Glioblastoma 70 (Loeffler) dataset, and “T H (84)”, representing the T Glioblastoma 84 (Hegi) dataset as the GBM tumor group. Boxplots were generated via R2: MegaSampler. Box limits indicate the range of the central 50% of the data (from 75th to 25th quantiles) with the central line marking the median value. The upper and lower whiskers are the maximum and minimum values of the data that are within 1.5 times the interquartile range over the 75th and 25th percentiles. *P*-values were computed via one way analysis of variance (ANOVA). Datasets of normal (N) samples are labeled in gray, and datasets of glioblastoma tumor (T) samples are labeled in salmon under the *x*-axis. The numbers in brackets represent the sample sizes in the datasets.

Next, the expression levels of the 105 DEGs were also analyzed using TCGA-GBM RNA-seq data, with 98 of these genes being identified from TCGA-GBM transcriptome profiling data. The results are summarized in Fig. 6a, with seven of the 10 candidate genes highlighted. In addition, due to the limited number of normal brain tissues we collected, the expression of *MMP9*, *TMEM92*, *C1orf226*, *CD163*, *LINC00482*, *AK5*, *MICU3*, and *CD200* in R2 tumor tissue data was also analyzed using the R2 online tool to validate our results and is shown in Fig. 6b. Taken together, ten candidate blood markers, including the expression signature of seven mRNAs, one lncRNA and two miRNAs, were identified for GBM and designated the “GBM-Dx” panel.

## Discussion

In this study, by analyzing a total of 47 samples, including blood and tumor specimens derived from 36 donors, including non-GBM individuals and GBM patients, and external data from a total of 576 tissue samples, including 221 normal or nontumor tissues as controls and 355 tumor tissues (Table 1), we present WBGR as a useful and advantageous approach for whole blood-based GBM biomarker mining. Its key merits in blood transcriptome analysis include the improvement of detection sensitivity, the efficient enrichment of informative reads towards DGE analysis, and the retention of high expression concordance of non-globin genes after GR. These values allowed us to comprehensively analyze DGE in GBM blood and to distinguish blood samples of GBM patients from those of non-GBM donors by a list of DEGs.

**Table 1:** Tabular summary of the demographic and clinical characteristics of patients with GBM and control subjects.

	Control	GBM
Blood for RNA-seq		
Patient number (*n*)	12 (healthy, parkinsonism, pseudotumor cerebri, leptomeningeal disease, pancreatitis, chronic headaches, peripheral neuropathy, or chronic prostatitis, etc.)	10
Age^a^ in years, mean ± SD	51.8 ± 16.5	57.3 ± 10.9
Sex, male, *n* (%)	7 (58.3%)	7 (70%)
Cancer stage *n* (%)	N/A	WHO-IV (100%)
Recurrent, *n* (%)	N/A	6 (60%)
Treatment before blood draw, *n* (%)	N/A	8 (80%)
Blood for RT-qPCR		
Patient number (*n*)	2 (healthy)	5
Age^a^ in years, mean ± SD	52.5 ± 5.5	48.6 ± 17.8
Sex, male, *n* (%)	1 (50%)	2 (40%)
Cancer stage *n* (%)	N/A	WHO-IV (100%)
Recurrent, *n* (%)	N/A	2 (40%)
Treatment before blood draw, *n* (%)	N/A	5 (100%)
Tissues		
Patient number (*n*)	2 (normal adjacent tissues of 2 GBM patients)	10
Age^a^ in years, mean ± SD	40.5 ± 19.5	50.1 ± 13.3
Sex, male, *n* (%)	2 (100%)	6 (60%)
Cancer stage *n* (%)	N/A	WHO-IV (100%)
Recurrent, *n* (%)	N/A	5 (40%)
Treatment before tissue excision, *n* (%)	N/A	2 (20%)
TCGA tissues (transcriptome profiling data)		
Patient number (*n*)	5 (normal solid tissues)	156
Age^b^ in years, mean ± SD	N/A	59.9 ± 13.6
Sex, male, *n* (%)	N/A	99 (64.7%)
Cancer stage *n* (%)	N/A	WHO-IV (100%)
Recurrent, *n* (%)	N/A	0 (0%)
R2 tissues (array RNA profiling datasets)		
Patient number (*n*)	216 (nontumor or normal brain tissues)	200
Age^b^ in years, mean ± SD	N/A	N/A
Sex, male, *n* (%)	N/A	Percentage N/A
Cancer stage *n* (%)	N/A	WHO-IV (100%)
Recurrent, *n* (%)	N/A	Percentage N/A

*N/A* not applicable, not available, or not reported.

^a^Age at procedure.

^b^Age at diagnosis.

We have previously demonstrated an increased sensitivity to discern the difference between three males and eight females by GR in a blood microarray assay^24^. Shin et al. subsequently showed the technical and biological benefit of GR in the RNA-seq transcriptome profile of human peripheral blood^22^. GR is a critical step for blood RNA-seq, as both previous and current studies show that it improves the accuracy of detection and increases the sequencing capacity of informative RNA reads. Abundant hemoglobin-related RNAs may increase the amount of noise for subsequent sequencing after an amplification step of RNA samples. In Mastrokolias et al.’s study^25^, they found the expression of over 2000 transcripts in globin-reduced samples that could not be detected in non-globin-reduced samples, suggesting that transcripts are masked by high amounts of hemoglobin transcripts. Based on our previous work^24^, our experience, the advancement of sequencing technology, and the limited human GBM sample availability, we considered that 10 whole blood samples from patients with GBM versus 12 whole blood samples from individuals without GBM in blood RNA-seq is appropriate for the discovery phase. Indeed, we have made the comparison and obtained significant results. In addition, we included datasets from the publicly available databases TCGA and R2 as described above. Our results show that GR prior to RNA sequencing library preparation significantly increased the globin noise reducing index N_G_RI scores, improved the overall informative reads of the sequencing data, and hence resulted in the detection of more DEGs from this study (Fig. 2f). With WBGR, we identified significantly changed genes in the blood of a GBM cohort and mapped out the most GBM-related pathways reflected in blood by Reactome pathway analysis as well as Baylor Modular Analysis enriched immune-related genes potentially functioning in GBM. Reactome is an open access, manually curated, and peer-reviewed pathway database, while our Baylor Modular Analysis offers a means to illustrate the immunological mechanisms relevant to human diseases on a genome-wide scale and provide a foundation for the discovery of clinically relevant biomarker signatures^32,35^. Based on the literature, two other studies^17,40^ used samples from normal healthy or non-GBM individuals as controls. This study included age- and sex-matched healthy individuals and nontumor patients in the control group, which is in line with other studies. Thus, the integrated protocol we used in this work can be applied in blood biomarker discovery for a broad range of disease conditions, including other types of cancer and other brain disorders.

By a comprehensive analysis of the post-GR blood RNA-seq data and subsequent validation in independent blood and tumor tissue samples, we identified a GBM-Dx panel (ten gene candidates, including seven mRNAs, one lncRNA, and two miRNAs) as a biomarker signature distinguishing GBMs from non-GBM controls. Specifically, *MMP9*, *TMEM92*, *C1orf226*, *CD163*, *LINC00482*, and miR-3918 are upregulated, and *AK5*, *CD200*, *MICU3*, and miR-760 are downregulated in GBM. Among them, *MMP9* and *CD163* are known players in GBM development. Importantly, successful identification of these known players in GBM validated our approach to identifying novel genes such as *C1orf226*, *TMEM92*, *AK5*, *MICU3*, and miR-3918 that may be implicated in the development of GBM as new players. Looking at the functions reported for the known players, highly expressed *MMP9* in GBM contributes to cancer proliferation and progression^41,42^; *CD163* is involved in the regulation of glioma cell stemness and proliferation^43^; and co-overexpression of *CD163* and *MMP9* may indicate the polarization of macrophages associated with tumor cells^44^, supporting their potential to serve as GBM blood markers together. In addition, from our results, a variety of DEGs have not been previously reported to be relevant to GBM, and their functions are largely unknown, such as *LINC00482*, *TMEM92*, *C1orf226*, miR-3918, and miR-760. The elevated level of *LINC00482* in the GBM group may be both a novel potential detectable biomarker and a therapeutic target. According to analysis of TCGA and R2 genomics datasets, a higher expression of *C1orf226* in tumor tissue is observed in GBM tumors in accordance with our results shown in Figs. 4, 5. miR-3918 expression has been observed in the culture medium of medulloblastoma (MB) cell lines and MB patient CSF, but our study is the first to show that a high level of miR-3918 is associated with GBM. Previous studies have reported miR-760 downregulation in various cancer types, such as colon cancer, lung cancer, and breast cancer^45^. Iwaya et al. reported that by directly interacting with histone mRNAs, miR-760 downregulation occurs in both the bone marrow and primary tumors of advanced gastric cancer patients^46^. Our results show that miR-760 expression is also decreased in GBM patients. In the case analyses (Fig. 5c), genes such as *TMEM92* and *C1orf226* showed consistent expression in pretreatment surgically resected tumor tissues, and posttreatment blood may be indicators of GBM. Genes such as *AK5* and *MICU3* showed expression changes in pretreatment tumor tissue, and posttreatment blood may be indicators of treatment response. Functional studies of these genes in the context of GBM may provide deeper insights into GBM pathology.

In summary, we developed and tested a robust and sensitive methodology, WBGR, for whole blood-based profiling of transcriptomic traits of GBM. By using WBGR, we successfully characterized a 10-gene GBM-Dx panel representing the GBM blood signature, which may be developed as a clinical tool such as a patient-specific signature barcode. To further expand the potential clinical applications of the GBM DEGs identified in this work, additional knowledge of the detection sensitivity and specificity is desirable. Our approach and the identified GBM-Dx panel await (1) further validation in a larger patient cohort, which may include control patients with other cancers, to allow clinical application in the coming years, (2) further integration with genetic alteration detection (for example, *EGFR* alterations, *MGMT* methylation, and alterations in genes reported in the GBM-Dx panel) and tumor tissue transcriptomic subtypes (e.g., classic, mesenchymal-like) to provide an expanded view of the intrapatient tumor molecular characteristics and interpatient heterogeneity in blood, and (3) further complementation with individual neuroimaging metrics to enable patient stratification and prognostic modeling for the efficient and effective management of patients with GBM.

## Methods

### Human peripheral blood samples

Human peripheral blood samples were collected at Baylor Scott & White Medical Center-Temple (BSW-Temple), Temple, Texas, USA. Permission to collect human whole peripheral blood was obtained from the institutional review board (IRB) at Baylor Scott & White Health. Written informed consent forms for the use of blood samples and accompanying clinical information for research purposes were obtained from all the participants. The study was performed under IRB-approved guidance and regulations to keep all patient information strictly deidentified. The inclusion criteria were as follows: (i) adult patients diagnosed with GBM (de novo or secondary) were includable, (ii) patients with noncancer comorbid conditions such as hypertension, headache, obesity, and kidney diseases were acceptable, and (iii) adults without cancer diagnosed were acceptable as non-GBM controls. The exclusion criteria were (i) patients with other types of cancer or brain metastasis and (ii) patients with infectious diseases. There was no exclusion defined based on the sex, race or ethnicity of the subjects. In total, 15 GBM patients (six female) with a mean age of 54.4 years and 14 age- and sex-matched non-GBM donors (six female) with a mean age of 51.8 years provided samples. A summary of all subjects studied in this work is provided in Table 1. Detailed information for each participant, including age, sex, treatment status, recurrence status and vital status, is provided in Supplementary Table 1.

### Human brain tissue samples

Brain tumor tissue and normal adjacent samples were obtained from the Brain Bank at BSW-Temple. Permission to use human brain materials from BSW-Temple Brain Bank was obtained from the IRB at Baylor Scott & White Health. Only samples from patients who underwent surgical resection in their treatment plan were collected. Written informed consent forms for the use of brain tissue samples and accompanying clinical information for research purposes were obtained from all the participants. Patient information was strictly deidentified. In total, 10 GBM patients (10 tumor tissues and 2 normal adjacent samples obtained) were included with a mean age of 50.1 years (range 21–71 years) and 4 female patients. Three patients provided both blood and tumor tissues. Detailed information for each patient, including tissue type, age, sex, treatment, recurrence, vital status and comorbidity information, is provided in Supplementary Table 2.

### Globin reduction and blood RNA-seq

Blood samples collected at BSW-Temple were subjected to globin depletion, including non-GBM control blood (13 samples) and GBM blood (10 samples). Two milliliters of peripheral blood from participants was drawn with a BD safety-Lok^TM^ blood collection set (BD) into a PAXgene^TM^ RNA collection tube (Qiagen) and then kept at −80 °C. Total RNA, including small RNAs, was isolated with the PAXgene^TM^ Blood miRNA Kit (Qiagen). No specific step was performed to remove ribosomal RNAs during blood RNA isolation. The GLOBINclear^TM^ Kit (Ambion) was employed to remove the highly abundant hemoglobin mRNAs from the blood isolated RNA samples. Briefly, one microgram of total RNA from each sample was hybridized with a biotinylated capture OLIGO Mix (biotinylated oligonucleotides), which is specific for human hemoglobin mRNAs^25^. Streptavidin magnetic beads were added to bind the biotinylated oligonucleotides that hybridized with globin mRNAs, and the remaining RNAs were then pulled down by a magnet. The globin-depleted RNAs were further purified with a rapid magnetic bead-based purification process, and OLIGO Mix was removed. RNA concentration was assessed using a NanoDrop Spectrophotometer (NanoDrop Technologies). The resulting samples were stored at −80 °C before use.

RNA samples both pre- and post-GR with an RNA integrity number (RIN) ≥ 7.0 were used for cDNA paired-end library preparation with poly(A) selection. First, mRNAs and RNAs with poly(A) tails (such as some long noncoding RNAs) were purified from total RNA using oligo (dT)-attached magnetic beads and were then fragmented. First-strand cDNAs were generated using random hexamer-primed reverse transcription, followed by second-strand cDNA synthesis. Then, PCR was performed, PCR products were purified with AMPure XP beads (Agencount, Beckman Coulter), and library quality was validated on the 2100 Bioanalyzer system (Agilent). The double-stranded PCR products were heat denatured and circularized by the splint oligo sequence. Single-stranded circular DNA (ssCir DNA) was formatted as the final library. Libraries for mRNA-seq were sequenced on the Illumina HiSeq 2000 System at Beth Israel Deaconess Medical Center (BIDMC) Genomics Proteomics Core at Harvard Medical School, and 100 base pairs (bp) paired-end reads were generated. Small RNA library construction and sequencing were performed at BIDMC Genomics Proteomics Core. Briefly, approximately one microgram of RNA with an RIN ≥ 7.0 from each sample was used. RNA segments of different sizes were separated by PAGE, and 18–30 nucleotide stripes were selected and recycled. Then, a 3ʹ adaptor connection system, RT primer addition and 5ʹ adaptor connection system were prepared. After that, strand cDNA synthesis, PCR amplification and library fragment selection were performed. The double-stranded PCR products were heat denatured and circularized by the splint oligo sequence. ssCir DNA was formatted as the final library. Libraries for small RNA-seq were sequenced on a BGISEQ-500 platform at BIDMC Genomics Proteomics Core.

### Quality control and computational pipeline of RNA-seq data analysis

mRNA-seq generated an average of ~43.7 million high-quality clean reads per sample. Small RNA-seq yielded an average of ~23.05 million high-quality clean reads per sample. Sequencing data were processed using a quality control-human reference genome alignment-differential gene expression pipeline. The quality characteristics of sequence reads for each sample were over 97%. The Q20 for each sample was over 97%, and the GC contents were ~49–59% for the sequenced samples. A total of 97.6% of the clean reads from mRNA-seq and 89% of the clean reads from small RNA-seq were mapped to the human genome. Sequencing data were processed using a quality control-human reference genome alignment-differential gene expression pipeline (Supplementary Fig. 2) as follows. Quality control for raw sequencing reads was performed by FastQC^47^. Reads from mRNA-seq were aligned to the GRCh38 reference genome using hisat2^48^, and quality control metrics were calculated using the Picard toolkit^49^. mRNA-seq samples with less than 30% of the reads mapping to coding regions were removed. SAM files obtained from the aligner were converted to BAM format using SAMtools^50^. FeatureCounts^51^ was used to quantify the total number of counts for each gene. edgeR^52^ was used to generate counts-per-million values. Genes with more than one read counts per million in at least two samples were kept for downstream analysis. The small RNA sequencing reads were aligned and counted using the extracellular RNA processing toolkit (exceRpt)^53^. The pipeline first filtered the reads that mapped to the UniVec vector and ribosomal RNA sequences, and the unmapped reads were then aligned to the human genome (GRCh38) and quantified for different types of RNAs, including miRNAs (miRBase v21 [http://www.mirbase.org/]) and other small noncoding RNAs. Principal component analysis was performed using the ggfortify R package^54^. The globin noise reduction index (N_G_RI) for each sample was defined as follows:

1 $$N_GRI = \frac{{1 - N_G}}{{N_G}}$$

2 $$N_G = \mathop {\sum}\nolimits_{i = 1}^n {\alpha _i}$$

where α_i_ is the percentage of counts for each hemoglobin gene in a given sample and n is the total number of hemoglobin-related genes (*n* = 15 according to Ensembl^55^). A higher score represents a higher proportion of non-globin gene reads.

### Differential gene expression (DGE) analysis

DGE analysis was performed between the GBM group and the control group for both mRNA-seq and small RNA-seq data using the DESeq2 package in R^56^. Sequencing read counts were normalized using the median-of-ratios method^57^ and logarithmic base 2 (log2) transformed for data visualization. Normalized mRNA count distributions in all the samples are shown in Supplementary Fig. 3. Genes that survived FDR ≤ 0.05 or *p*-value ≤ 0.05 were considered differentially expressed, i.e., DEGs. Gene expression heatmaps were plotted using the ComplexHeatmap package^58^ in R. Hierarchical clustering of representative mRNA and miRNA expression was also performed.

### miRNA-target analysis

Blood DEMs (33 miRNAs) were analyzed for their gene targets. Predicted gene targets of the DEMs from multiple miRNA-target databases, e.g., miRecords, miRTarBase, and TarBase, were retrieved using the multiMiR R package^30^, and only target genes that were validated by experiments were selected.

### External datasets

We downloaded RNA-seq data of GBM tumor tissues and solid tissue normal samples from The Cancer Genome Atlas (TCGA) (https://www.cancer.gov/tcga). Transcriptome RNA profiling data available for 156 primary GBM tissues reported in Brennan et al.’s paper^31^ and 5 solid normal tissue controls^33^ were extracted and analyzed for differential gene expression. The case IDs of the data used in this study are provided in Supplementary Table 3. DGE analysis was performed between the GBM tumor group and the solid normal group using DESeq2; genes that survived FDR ≤ 0.05 were considered differentially expressed. The generated gene list was compared with blood RNA-seq data to identify overlapping genes.

We also queried R2: Genomics Analysis and Visualization Platform (http://r2.amc.nl)^59^ for DGE analysis of GBM tissues compared with normal brain tissues. Two datasets of normal brain tissues (“Normal Brain PFC - Harris - 44 - MAS5.0 - u133p2”^60^ and “Normal Brain regions - Berchtold - 172 - MAS5.0 - u133p2”^61^) and three datasets of GBM tumor tissues (“Tumor Glioblastoma - Hegi - 84 - MAS5.0 - u133p2”^62^, Tumor Glioblastoma - Loeffler - 70 - MAS5.0 - u133p2”^63^ and “Tumor Glioblastoma - Pfister - 46 - MAS5.0 - u133p2”^64^) were selected and analyzed through R2: megasearch online portal. Gene expression differences were considered significant at FDR ≤ 0.01. The generated DEGs were also compared with blood RNA-seq data to identify overlapping genes. Heatmaps were plotted using the ComplexHeatmap package^58^ in R. Boxplots of analyzed R2 datasets were generated on the R2 website.

### Reactome pathway analysis

DEGs from tissue RNA-seq data and blood RNA-seq data were used for functional analysis, which was performed with the Reactome Pathway Database (https://reactome.org/)^32^. Annotated Reactome pathways with FDR (q-value) ≤0.1 were considered significantly enriched and are shown by dot plots generated using the ggplot2 package^65^ in R. The network showing major pathways included in both blood data and tissue data was visualized using the igraph package^66^ in R.

### Baylor Modular Analysis

To facilitate interpretation of the gene expression signature, we used a pre-existing framework of 260 transcriptional modules, including more than 14,000 transcripts specific to blood samples collected from a wide range of diseases, established by Chaussabel et al. previously at our institution to analyze this dataset, enriching immune-related genes associated with GBM^34,35^. For each module, the percentage of transcripts significantly up- or downregulated was calculated, and the module score was defined as the difference in percent up or down, designated as the proportion. If a module in which x% transcripts are significantly upregulated and y% transcripts are significantly downregulated, the module score would be x − y. Proportion values of

$${{{\mathrm{x}}}}-{{{\mathrm{y}}}} \,>\, 0\;{{{\mathrm{or}}}}\;{{{\mathrm{x}}}}-{{{\mathrm{y}}}} \,<\, 0$$

are represented by red or blue spots, respectively. Data were considered significant at FDR ≤ 0.05 and visualized using the ComplexHeatmap R package^58^. This approach can detect small but codependent changes in transcripts that may not be considered to be significant when analyzing each gene as an independent variable^34^.

### Real-time quantitative PCR (RT-qPCR) analysis

Total RNA, including small RNAs, was isolated from tumor tissues (12 in total) and blood of GBM patients (5 independent samples + 4 sequenced samples) or control counterparts (3 independent samples + 2 sequenced samples) using the miRNeasy Mini Kit (Qiagen) and PAXgene^TM^ Blood miRNA Kit (Qiagen), respectively. For brain tissue specimens, total RNA, including small RNAs, was isolated using the miRNeasy Mini Kit (Qiagen). For miRNA detection, total RNA was used to complete reverse transcription and PCR with the miScript PCR system, including the miScript II RT Kit, miScript Primer Assays and miScript SYBR Green PCR Kit (Qiagen). For mRNA detection, total RNA was used to prepare cDNA using the iScript Reverse Transcription Supermix (Bio-Rad) and then subjected to qPCR assay. RT-qPCR was performed using iTaq Universal SYBR Green Supermix (Bio-Rad). All qPCR assays were performed in triplicate on a CFX96 Touch™ Real-Time PCR Detection System. Cycle threshold (Ct) values were calculated using the automated settings of the system. Fold change (FC) obtained from Ct values using 2^−ΔΔCt^ methodology^67^ was converted into log2 for statistical analysis. Human GAPDH, U6 and 18S rRNA were used as controls for PCRs of mRNAs, miRNAs and long noncoding RNAs (lncRNAs), respectively. Gene-specific primers were designed using the Primer-BLAST online tool^68^. Genes whose primers generate amplicons with expected lengths according to the primers used and show a single band on a gel were tested via qPCR. The primer sequences are listed in Supplementary Table 4. Comparisons were made between the GBM group and the control group. qPCR data summary and data from three blood and matched tumor tissue pairs were visualized using boxplots and stacked bar charts plotted by the ggplot2 package in R. Multiple experimental results for each sample are presented as the mean ± standard deviation (SD) in Supplementary Fig. 4.

### Statistics

For statistical analyses, we used R (version 4.1.0, 4.1.2, and 4.2.1), R studio (version v1.3.1073, v1.4.1717, v2022.02.3 + 492, and v2022.07.1 + 554) and R packages such as ggpubr and rstatix^69,70^. The level of significance for gene expression differences between GBM and non-GBM control groups was analyzed by the moderated *t*-statistic in dataset analysis for RNA-seq data using the DESeq2 package in R or *t*-test for RT-qPCR data in R. Statistics for the Reactome pathway analysis were generated from their online portal. Statistical significance was defined as FDR ≤ 0.05 or *P*-value ≤ 0.05, unless otherwise specified.

### Reporting summary

Further information on research design is available in the Nature Research Reporting Summary linked to this article.

## Supplementary information

Supplementary file

Reporting Summary

## Data Availability

Data generated from this study have been included in the manuscript files. Publicly available databases used in this study have been cited, and detailed information has been provided in this manuscript. The sequencing count data of this study are available upon request to E.W. or at GitHub (https://github.com/ddqq666/gProj), and the raw data were deposited into the NCBI Sequence Read Archive (SRA) database (BioProject ID: PRJNA878767).

## Code availability

All codes used to perform the analysis are available upon request to E.W. or at https://github.com/ddqq666/gProj. Key software and R packages used for data processing and visualization are described in the Methods section or figure legends.
