# Supplementary material for: Transcriptomic analyses of patient peripheral blood with hemoglobin depletion reveal glioblastoma biomarkers
Source: NPJ Genom Med. 2023 Jan 25;8:2. doi: 10.1038/s41525-022-00348-3 (PMC9877004; doi:10.1038/s41525-022-00348-3)
Supplement: Supplementary file 1 — Supplementary file [file 41525_2022_348_MOESM1_ESM.pdf]

Supplementary Materials

Transcriptomic analyses of patient peripheral blood with hemoglobin depletion reveal glioblastoma biomarkers

Dan Qi [1,10], Yiqun Geng [1,2,10], Jacob Cardenas [3], Jinghua Gu [3], S. Stephen Yi [4,5,6,7], Jason H. Huang [1,8,✉], Ekokobe Fonkem [1,✉], Erxi Wu [1,7,8,9,✉]

[1] Department of Neurosurgery and Neuroscience Institute, Baylor Scott & White Health, Temple, TX 76508, USA

[2] Laboratory of molecular pathology, Shantou University Medical College, Shantou 515041, China

[3] Baylor Scott & White Research Institute, Dallas, TX 75204, USA

[4] Institute for Cellular and Molecular Biology (ICMB), College of Natural Sciences, The University of Texas at Austin, Austin, TX 78712, USA

[5] Oden Institute for Computational Engineering and Sciences (ICES), The University of Texas at Austin, Austin, TX 78712, USA

[6] Department of Biomedical Engineering, Cockrell School of Engineering, The University of Texas at Austin, Austin, TX 78712, USA

[7] Department of Oncology, LIVESTRONG Cancer Institutes, Dell Medical School, The University of Texas at Austin, Austin, TX 78712, USA

[8] Texas A & M University School of Medicine, Temple, TX 76508, USA

[9] Texas A & M University School of Pharmacy, College Station, TX 77843, USA

[10] These authors contributed equally as first authors.

✉Corresponding Authors: Erxi Wu (lead contact), Erxi.Wu@BSWHealth.org; Ekokobe Fonkem, Ekokobe.Fonkem@BSWHealth.org; Jason H. Huang, Jason.Huang@BSWHealth.org.

Running title: GBM biomarker discovery using WBGR

Supplementary Note

List of Figures

Supplementary Fig. 1: Overview of the experimental workflow.

Supplementary Fig. 2: Overview of the computational pipeline.

Supplementary Fig. 3: Data filtering, normalization and principal component analysis.

Supplementary Fig. 4: RT-qPCR results in triplicate for all samples.

List of Tables

Supplementary Table 1: Clinical data table-blood.

Supplementary Table 2: Clinical data table-tumor tissue.

Supplementary Table 3: TCGA tissue transcriptome profiling data used in this study.

Supplementary Table 4: Primers used in PCR validation.

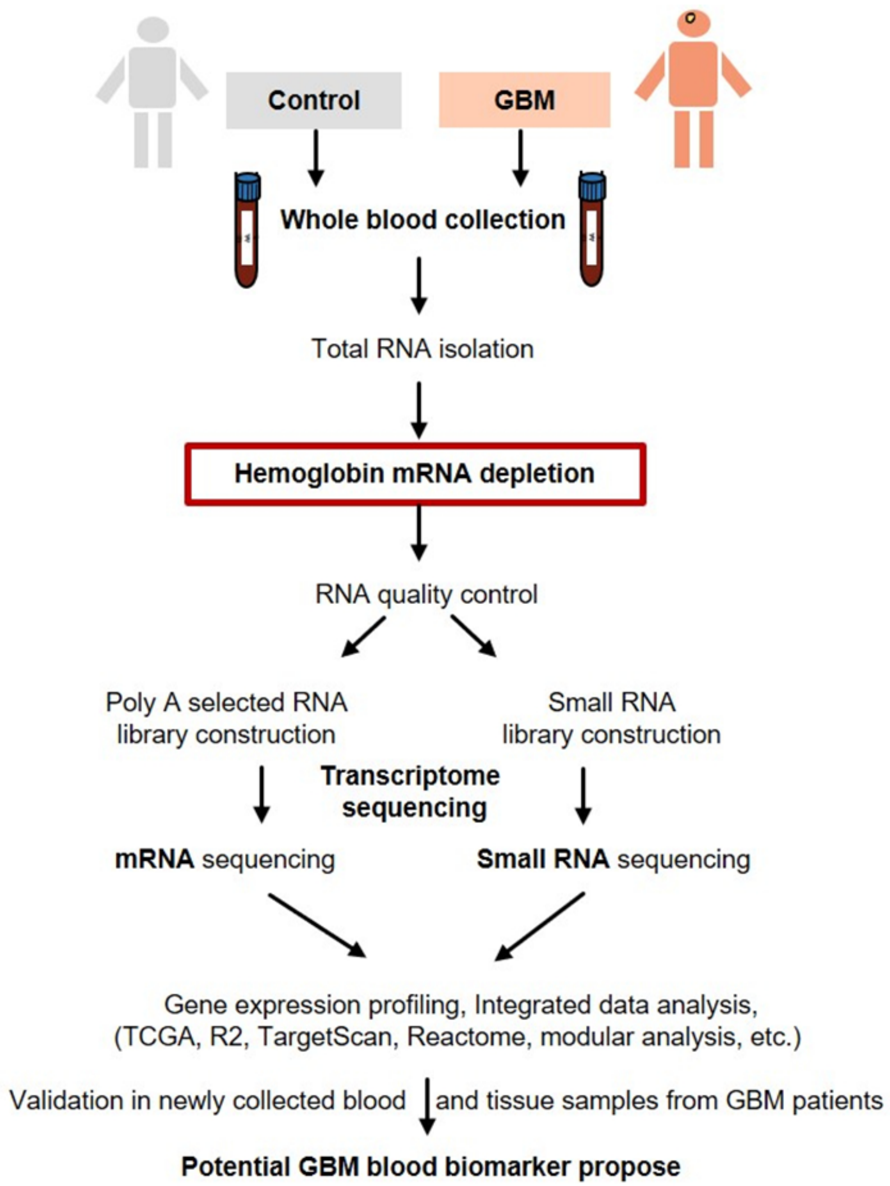

Supplementary Fig. 1| Overview of the experimental workflow.

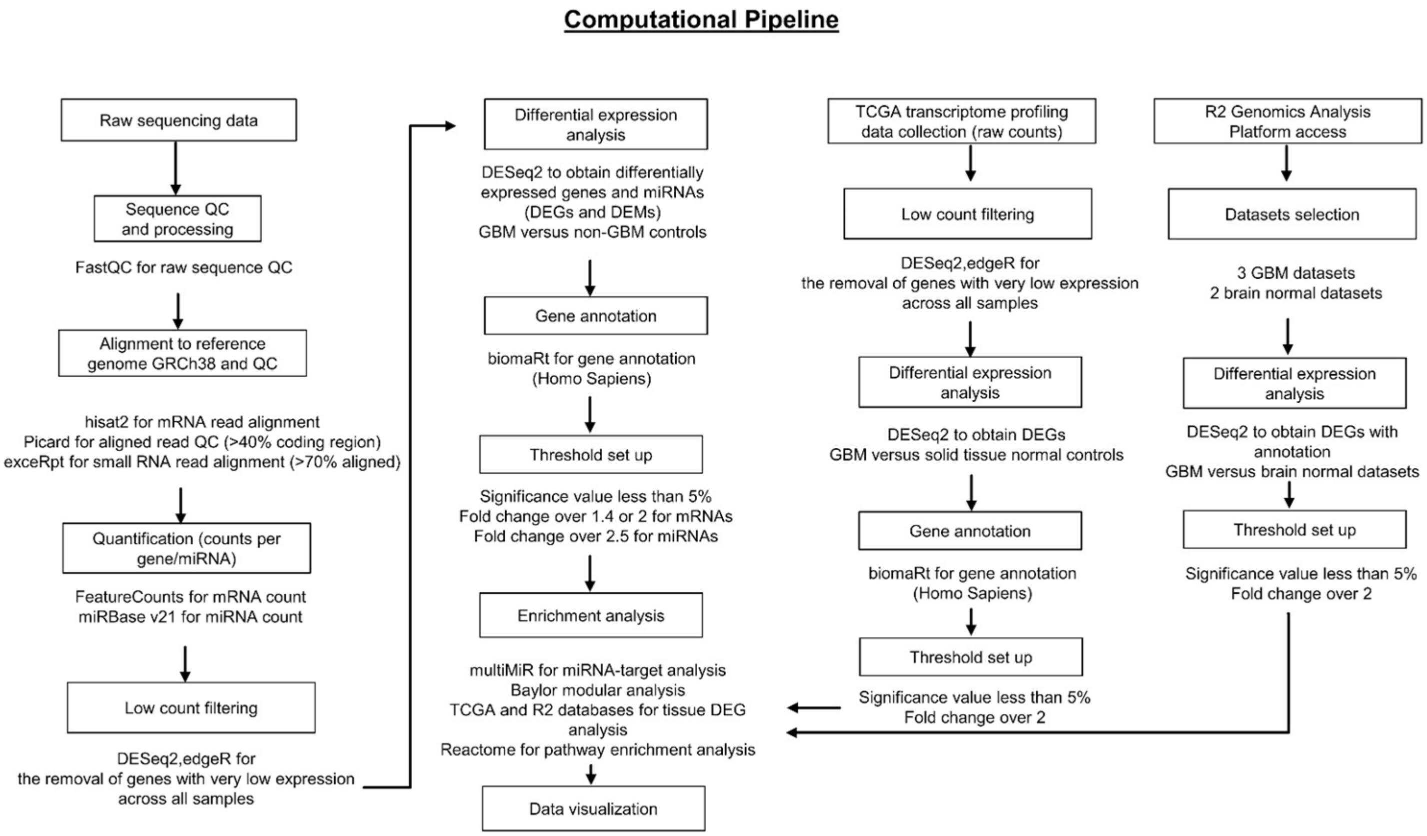

Supplementary Fig. 2| Overview of the computational pipeline.

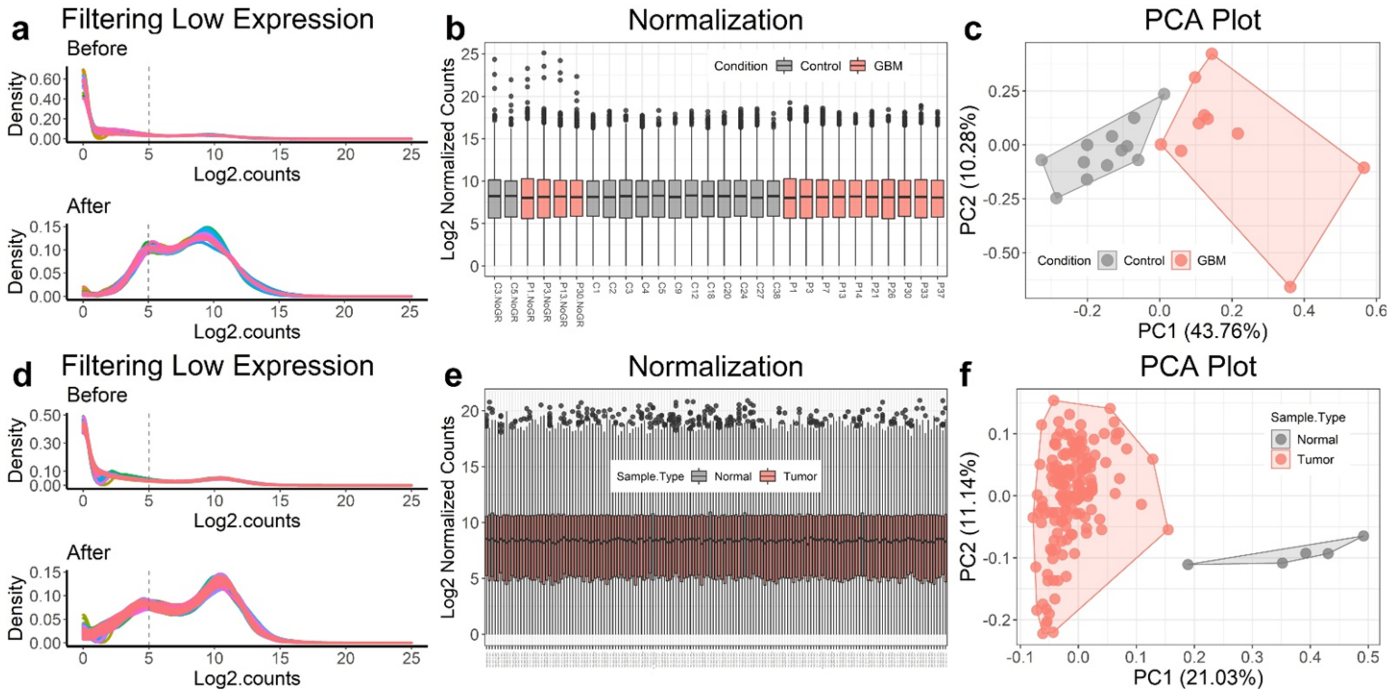

Supplementary Fig. 3| Data filtering, normalization and principal component analysis. a-c, Blood RNA-seq data check**. a**, Count filtering to remove transcripts with low counts in most samples. **b,** Count distributions across samples showing a similar pattern after log2 normalization. **c,** Principle component analysis (PCA) using normalized counts. **d-f,** TCGA RNA-seq data check. **d,** Count filtering to remove transcripts with low counts in most samples. **e,** Count distributions across samples showing a similar pattern after log2 normalization. **f,** Principle component analysis (PCA) using normalized counts.

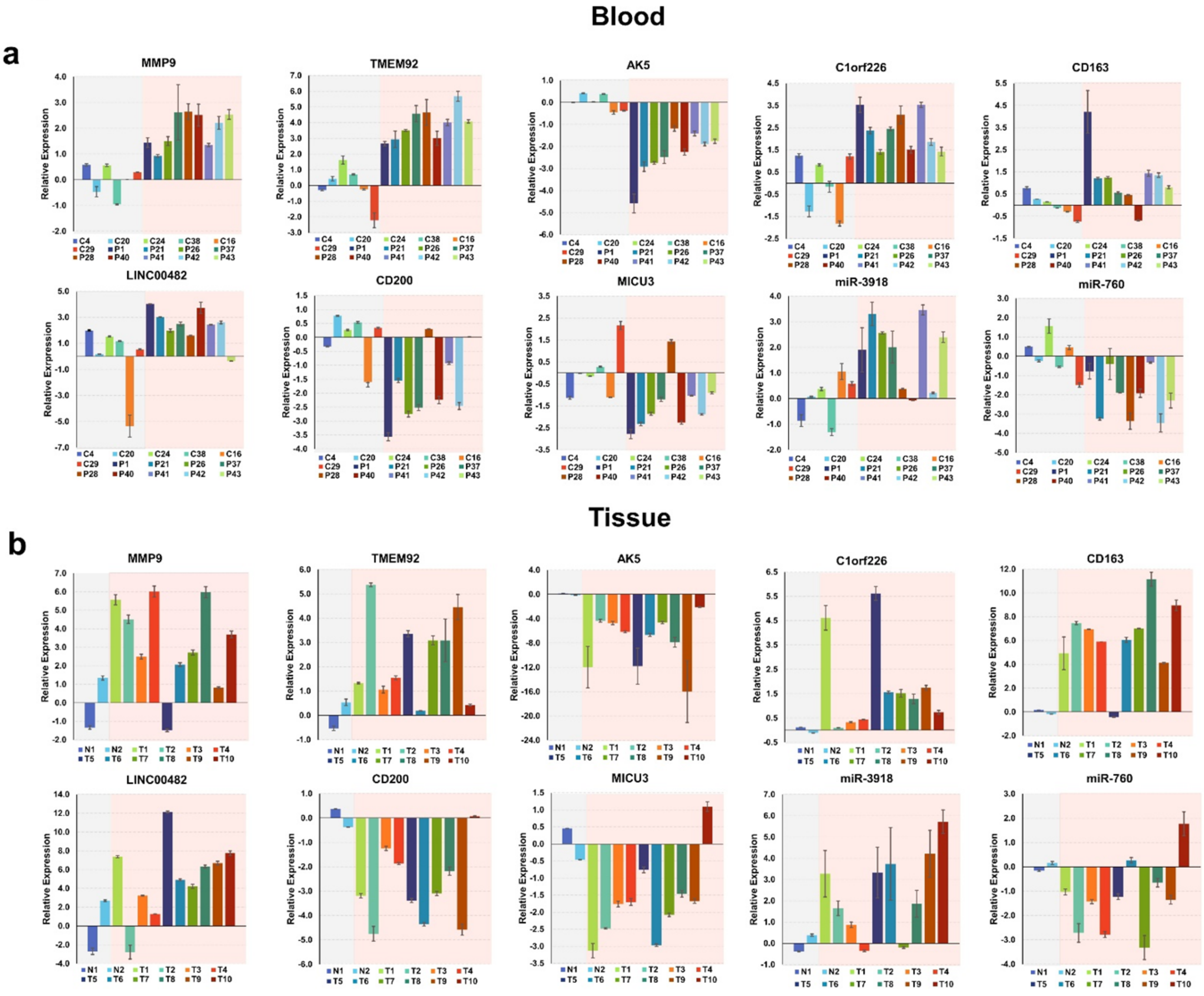

Supplementary Fig. 4| RT-qPCR results in triplicate for all samples. a, Triplicates of PCR results of blood samples summarized as the mean ± SD. **b,** Triplicates of PCR results of tumor tissue samples summarized as the mean ± SD. The background in light gray indicates data from the control (blood) or normal (tissue) groups, and the background in light red indicates data from the GBM (blood) or tumor (tissue) groups.

Supplementary Table 1. Clinical Data Table-Blood

Sample ID	Diagnosis	Age[a]	Race	Gender	Recurrent	Treatment[b]	Vital Status	Analysis	Note
C1	Control	27	White	Female	NA	NA	NA	mRNA-seq	
C2	Control	22	White	Female	NA	NA	NA	mRNA-seq, small RNA-seq	
C3	Control	65	White	Male	NA	NA	NA	mRNA-seq, small RNA-seq	
C4	Control	66	White	Female	NA	NA	NA	mRNA-seq, small RNA-seq, RT-qPCR	
C5	Control	71	White	Female	NA	NA	NA	mRNA-seq	
C9	Control	61	White	Male	NA	NA	NA	mRNA-seq, small RNA-seq	
C12	Control	61	White	Male	NA	NA	NA	mRNA-seq, small RNA-seq	
C18	Control	50	White	Male	NA	NA	NA	mRNA-seq	
C19	Control	31	White	Female	NA	NA	NA	mRNA-seq	Removed after QC
C20	Control	30	White	Male	NA	NA	NA	mRNA-seq, small RNA-seq, RT-qPCR	
C24	Control	69	White	Female	NA	NA	NA	mRNA-seq	
C27	Control	44	White	Male	NA	NA	NA	mRNA-seq	
C38	Control	54	White	Male	NA	NA	NA	mRNA-seq, RT-qPCR	
C16	Control	47	White	Female	NA	NA	NA	RT-qPCR	
C29	Control	58	White	Male	NA	NA	NA	RT-qPCR	
Mean age of control group		51.79							Removed sample not included
Sex, female number : male number				F6: M8					Removed sample not included
P1	GBM	72	White	Female	No	No	Deceased	mRNA-seq, RT-qPCR	
P3	GBM	66	White	Male	Yes	Yes	Deceased	mRNA-seq, small RNA-seq	
P7	GBM	58	White	Male	Yes	Yes	Deceased	mRNA-seq	
P13	GBM	42	White	Male	Yes	Yes	Alive	mRNA-seq	
P14	GBM	54	White	Male	No	Yes	Alive	mRNA-seq, small RNA-seq	
P21	GBM	57	White	Male	Yes	No	Deceased	mRNA-seq, small RNA-seq, RT-qPCR	
P26	GBM	37	White	Female	No	Yes	Alive	mRNA-seq, small RNA-seq, RT-qPCR	
P30	GBM	72	White	Male	Yes	Yes	Deceased	mRNA-seq, small RNA-seq	
P33	GBM	61	White	Male	No	Yes	Alive	mRNA-seq	

P37	GBM	54	White	Female	No	Yes	Alive	mRNA-seq, small RNA-seq, RT-qPCR
P28	GBM	57	White	Female	Yes	Yes	Alive	RT-qPCR
P40	GBM	59	White	Female	No	Yes	Alive	RT-qPCR
P41	GBM	70	White	Female	No	Yes	Deceased	RT-qPCR
P42	GBM	37	African America n (Black)	Male	Yes	Yes	Alive	RT-qPCR
P43	GBM	20	White	Male	No	Yes	Alive	RT-qPCR
Mean age of GBM group		54.4						
Sex, female number : male number				F6: M9				

a, age at lab draw (years);
b, treatment received or not before taking blood samples;
F, Female;
M, male;
QC, quanlity control;
NA, not available or not applicable.

Supplementary Table 2. Clinical Data Table-Tissue

Sample ID	Diagnosis	Age[a]	Race	Gender	Recurrent	Treatment[b]	Vital Status	Analysis	Note
N1	Normal adjacent tissue of T1	21	Hispanic	Male	Yes	Yes	Deceased	RT-qPCR	
N2	Normal adjacent tissue of T2	60	White	Male	Yes	No	Deceased	RT-qPCR	
T1	GBM	21	Hispanic	Male	Yes	Yes	Deceased	RT-qPCR	
T2	GBM	60	White	Male	Yes	No	Deceased	RT-qPCR	
T3	GBM	57	White	Male	Yes	No	Alive	RT-qPCR	P21
T4	GBM	50	White	Female	No	No	Deceased	RT-qPCR	
T5	GBM	57	White	Female	Yes	No	Alive	RT-qPCR	P28
T6	GBM	52	White	Male	No	No	Deceased	RT-qPCR	
T7	GBM	71	White	Male	No	No	Alive	RT-qPCR	
T8	GBM	36	White	Female	No	No	Alive	RT-qPCR	P26
T9	GBM	55	White	Female	No	No	Alive	RT-qPCR	
T10	GBM	42	White	Male	Yes	Yes	Alive	RT-qPCR	
Mean age of control group		50.1							
Sex, female number : male number				F4: M6					

a, age at procedure (years);

b, treatment received or not before sample collection.

F, Female;

M, male;

NA, not available or not applicable.

Supplementary Table 3.TCGA Tissue RNA-Seq data used in this study

Project.ID	Sample.ID	Sample. Type	File.Name
TCGA-GBM	TCGA-27-1830-01A	Tumor	c153c4d8-2005-440d-9249-e8b8f2f73a96
TCGA-GBM	TCGA-06-5408-01A	Tumor	37889f67-62a8-483b-97af-d8391806ede9
TCGA-GBM	TCGA-32-2632-01A	Tumor	64718111-dba7-4f49-9f3a-7f2bccc12cda
TCGA-GBM	TCGA-06-2557-01A	Tumor	f728fc58-a4b9-4525-971a-a74a7d676695
TCGA-GBM	TCGA-26-5135-01A	Tumor	da09dd77-0b91-4795-bd80-f90a9784b65c
TCGA-GBM	TCGA-06-0156-01A-2	Tumor	c341aa36-a431-477a-9fae-4550c4eea047
TCGA-GBM	TCGA-28-2513-01A	Tumor	42959bfe-1a62-465f-aafc-684d973b43f7
TCGA-GBM	TCGA-02-2486-01A	Tumor	38195109-056a-4a1f-997e-0f77e20b647b
TCGA-GBM	TCGA-19-1787-01B	Tumor	ac22ae42-0d53-4b2e-ad6c-f4c98f9be040
TCGA-GBM	TCGA-02-0055-01A	Tumor	2556ce90-db5f-4beb-897a-db3cf556e054
TCGA-GBM	TCGA-27-1837-01A	Tumor	39701843-3dae-4114-b04c-b3387e402e4f
TCGA-GBM	TCGA-27-2528-01A	Tumor	e9a0fc7b-94ea-477d-918a-1ef80fefefcd
TCGA-GBM	TCGA-08-0386-01A	Tumor	b0717300-0735-4451-bba1-7ce0fc0d4ac6
TCGA-GBM	TCGA-06-0178-01A	Tumor	6b015617-e989-4c20-b81f-15bf94d83a09
TCGA-GBM	TCGA-76-4932-01A	Tumor	284d5f4a-5b44-42f7-90e6-14a5250183aa
TCGA-GBM	TCGA-26-5133-01A	Tumor	c8460566-8199-4a4d-93fd-67692611992d
TCGA-GBM	TCGA-19-2619-01A	Tumor	95b626ff-eb72-4539-b85b-2dea5a174fc2
TCGA-GBM	TCGA-06-2558-01A	Tumor	fcc54ed3-5ba9-461f-a3a5-72b8eb89e4da
TCGA-GBM	TCGA-14-0817-01A	Tumor	2f7160fe-5f34-49bc-95fd-029ce83d8c42
TCGA-GBM	TCGA-19-2624-01A	Tumor	4612a611-2ff9-4f51-b4b2-af142254dbe8
TCGA-GBM	TCGA-32-1982-01A	Tumor	8b4a22e2-e19a-4f8c-9f8f-a19f69fc8cbf
TCGA-GBM	TCGA-06-2561-01A	Tumor	844bbccc-8e4e-4c0a-ad29-0aa196f14c26
TCGA-GBM	TCGA-28-5209-01A	Tumor	2a254376-7498-4c41-8c20-8b4bdd8369da
TCGA-GBM	TCGA-27-1835-01A	Tumor	89b84c10-956d-4ce6-8837-8d97db837d8c
TCGA-GBM	TCGA-27-2526-01A	Tumor	0e30bd18-8e8b-4c52-aace-b5587c6df51a
TCGA-GBM	TCGA-76-4926-01B	Tumor	760c3a6c-809b-4d7e-9ec6-f5b713e8057d
TCGA-GBM	TCGA-06-5416-01A	Tumor	d638d8cf-7276-49aa-b2f7-81eb10e7b33d
TCGA-GBM	TCGA-32-2638-01A	Tumor	0f12d09f-f512-4a6a-95e2-c8942488f2ff
TCGA-GBM	TCGA-28-5216-01A	Tumor	ea50d2da-4f84-46dc-8036-c34392793aed
TCGA-GBM	TCGA-06-0130-01A	Tumor	a5525401-79be-4c7a-9107-71c022778d65
TCGA-GBM	TCGA-06-2565-01A	Tumor	b3850d71-3e90-4b9f-9057-55297d8b72bc
TCGA-GBM	TCGA-06-0219-01A	Tumor	8a395eef-a9cc-4f21-b8d2-aefa292077cf
TCGA-GBM	TCGA-06-0129-01A	Tumor	a504b1cd-7d40-40e8-8d58-267c680768e5
TCGA-GBM	TCGA-14-1829-01A	Tumor	4d4a1165-e304-4aed-9efa-7f6f5bacdb5e
TCGA-GBM	TCGA-12-0616-01A	Tumor	6508275a-e712-424e-bdaf-b9e1b07b0f95
TCGA-GBM	TCGA-32-2616-01A	Tumor	f5bb4817-1727-41f9-b7f6-3a4e1818c2df
TCGA-GBM	TCGA-12-0619-01A	Tumor	3d4081b3-24c9-4dbd-a5fe-0fb29ce43737
TCGA-GBM	TCGA-06-5856-01A	Tumor	746ed118-eb9f-43a5-bcda-872999ae7128
TCGA-GBM	TCGA-06-0747-01A	Tumor	ca0a0a82-4e82-4559-9666-d19faa3edf8f
TCGA-GBM	TCGA-14-0781-01B	Tumor	9c14c8ea-c5c4-42b5-873b-231efb65898a
TCGA-GBM	TCGA-06-0644-01A	Tumor	2b421eac-4980-4b9c-bddd-ad0818ec15e6
TCGA-GBM	TCGA-06-2559-01A	Tumor	30e10839-2ba1-4fd1-82ed-ad8fe4e72794
TCGA-GBM	TCGA-19-2629-01A	Tumor	dc3875c2-1a88-491c-bf98-fa344dd3b07e
TCGA-GBM	TCGA-41-5651-01A	Tumor	7cc30f38-a19e-4552-a5ca-64a11575abed
TCGA-GBM	TCGA-28-5204-01A	Tumor	afe67ba6-d59f-4450-8fbd-624da67d47e5
TCGA-GBM	TCGA-28-5215-01A	Tumor	b25c8e4d-f6ca-4079-9120-b96147b9091e
TCGA-GBM	TCGA-41-2571-01A	Tumor	ce3cc39f-d031-4751-a9fc-d9c7b2fb168f

TCGA-GBM	TCGA-06-0184-01A	Tumor	21f38c14-7d7c-4156-8c85-88be68cae18d
TCGA-GBM	TCGA-06-0156-01A	Tumor	18fbf794-a85b-4b0d-9a5c-c1c0e0ede3d8
TCGA-GBM	TCGA-14-2554-01A	Tumor	d6cdbf9a-1009-4ee1-b041-a3744f4166ac
TCGA-GBM	TCGA-41-3915-01A	Tumor	2fcb9b43-8a11-4730-b57e-4e9fe9013f1c
TCGA-GBM	TCGA-32-5222-01A	Tumor	ab5f9e4e-ff6a-4353-83f4-5ffddca0050a
TCGA-GBM	TCGA-06-5417-01A	Tumor	3f171964-37b4-43a2-b2f9-1b51dd924a7e
TCGA-GBM	TCGA-14-0787-01A	Tumor	53d78258-1650-4793-a01d-d2c065215fb9
TCGA-GBM	TCGA-06-5859-01A	Tumor	020fcc42-97ff-4959-b52f-f7f119c3f643
TCGA-GBM	TCGA-12-3652-01A	Tumor	378f6079-6c4e-415b-8b5e-6c3c72e513b3
TCGA-GBM	TCGA-12-0618-01A	Tumor	dfa2ddfb-c161-4c8c-a538-be6e00a8466e
TCGA-GBM	TCGA-26-5132-01A	Tumor	c3b82e75-c8c5-4dbb-b227-9b54db04b754
TCGA-GBM	TCGA-06-0686-01A	Tumor	6836d774-e17b-4198-b22a-47d05819b05d
TCGA-GBM	TCGA-06-5412-01A	Tumor	19680925-a148-4f8a-af3b-9137746d663f
TCGA-GBM	TCGA-28-5207-01A	Tumor	456ef025-b7f0-4040-90dd-5bcc2e476bfc
TCGA-GBM	TCGA-06-5411-01A	Tumor	eb24e6f8-dc16-4a6d-b584-9046829cc2ef
TCGA-GBM	TCGA-19-5960-01A	Tumor	d984a0e2-d502-4003-bdbf-a0364d4e5a78
TCGA-GBM	TCGA-12-3653-01A	Tumor	820e3299-bd67-4ba8-a637-32d31205e120
TCGA-GBM	TCGA-14-1034-01A	Tumor	419b4c0a-c23f-4214-8de0-722d2ae0846b
TCGA-GBM	TCGA-06-0743-01A	Tumor	8cb39efc-4bfb-49df-bc48-159d9211bc5a
TCGA-GBM	TCGA-12-3650-01A	Tumor	06227615-f651-462b-948f-c305ba369efa
TCGA-GBM	TCGA-06-2564-01A	Tumor	48a2e1f7-04c0-4013-83b6-fde730168435
TCGA-GBM	TCGA-02-0047-01A	Tumor	9c10ea63-f990-4c9b-9149-df9583e59610
TCGA-GBM	TCGA-06-0141-01A	Tumor	29f824b7-d899-40c1-b53b-edcb3f8e3d2e
TCGA-GBM	TCGA-26-5134-01A	Tumor	600932fb-d31e-4b69-afe7-e3803513d71f
TCGA-GBM	TCGA-32-1980-01A	Tumor	bdba30ab-5b63-4e14-b203-8a834022db68
TCGA-GBM	TCGA-06-0646-01A	Tumor	44ed61ae-e74e-40d7-98ce-7803e040567f
TCGA-GBM	TCGA-14-0871-01A	Tumor	2fb19a94-bd55-4041-994a-082cd30746f3
TCGA-GBM	TCGA-16-0846-01A	Tumor	37355f52-90e5-43e8-a473-0d3bad45a394
TCGA-GBM	TCGA-06-0138-01A	Tumor	76c6e110-9a94-4fcf-a534-a21ba4698f86
TCGA-GBM	TCGA-06-5410-01A	Tumor	eaff8d68-43f9-48bf-ad8b-8d0f452d3775
TCGA-GBM	TCGA-76-4927-01A	Tumor	c3f677f7-168a-414b-8006-a9cec7da0994
TCGA-GBM	TCGA-06-0125-01A	Tumor	28f0c53a-abdd-4789-adcf-f59132ae3a92
TCGA-GBM	TCGA-28-2514-01A	Tumor	b38a841a-f57e-4170-8cbc-17c54aacd580
TCGA-GBM	TCGA-06-0745-01A	Tumor	bcdc68db-c874-4097-9c46-b06e331caaf5
TCGA-GBM	TCGA-28-1753-01A	Tumor	ed9fe516-13b7-45d1-8d17-40ca9a96bbd0
TCGA-GBM	TCGA-76-4931-01A	Tumor	f47c3aa6-0f0d-4e0d-b6cf-bf565d511d6d
TCGA-GBM	TCGA-27-1832-01A	Tumor	7f7121ff-1399-40da-a792-b7c31bc76570
TCGA-GBM	TCGA-06-0158-01A	Tumor	47b09db5-8cbf-4385-9178-d943b48ebc53
TCGA-GBM	TCGA-06-0139-01A	Tumor	a3e5cda5-f468-48f3-92fa-864b8bbbecdc
TCGA-GBM	TCGA-76-4925-01A	Tumor	2afdb646-75ca-4bc9-9c12-30a27f994ecd
TCGA-GBM	TCGA-28-5218-01A	Tumor	bcbb79d8-1d4a-4fbb-b16c-4df86839773e
TCGA-GBM	TCGA-15-0742-01A	Tumor	02d43414-c8c9-42d3-8009-f9fb164dc8e0
TCGA-GBM	TCGA-06-2562-01A	Tumor	8af78255-e426-4a5d-a997-84946f5e459c
TCGA-GBM	TCGA-06-0878-01A	Tumor	cd925805-378c-4070-a7f3-ba8084efd836
TCGA-GBM	TCGA-28-5220-01A	Tumor	5cb8a505-e571-4faa-ab14-71e7b6a45a89
TCGA-GBM	TCGA-14-1823-01A	Tumor	6a7c7af6-b3b4-44a1-93bb-79a143db53d0
TCGA-GBM	TCGA-28-1747-01C	Tumor	f1f609a2-4fd6-48fc-8a29-3bc39c6ff178
TCGA-GBM	TCGA-12-0821-01A	Tumor	3fca2c7c-2541-418d-8f45-2a48814ca574
TCGA-GBM	TCGA-06-0750-01A	Tumor	de55dea1-658a-4a84-ae5c-fc554e9c4213
TCGA-GBM	TCGA-32-2634-01A	Tumor	bd4b6975-90d9-43f8-aadc-344d04644822

TCGA-GBM	TCGA-28-2510-01A	Tumor	2db015e3-bc3d-4884-b306-9283d6799d17
TCGA-GBM	TCGA-27-1834-01A	Tumor	7c03de7a-fafa-4f25-96bd-dc283674a273
TCGA-GBM	TCGA-06-0749-01A	Tumor	fe09042c-c233-4f10-b8ad-d5da80aaf60d
TCGA-GBM	TCGA-06-5418-01A	Tumor	68fe871b-c631-469e-87a2-26130f416446
TCGA-GBM	TCGA-41-4097-01A	Tumor	681955cd-356d-4e64-924b-b0381e3c12bc
TCGA-GBM	TCGA-06-0211-01B	Tumor	f67cf68d-56b3-451d-ba21-566664213691
TCGA-GBM	TCGA-76-4929-01A	Tumor	502fc9a8-6409-4b7a-a0f8-abed308ce84a
TCGA-GBM	TCGA-27-2519-01A	Tumor	21800024-cf76-4185-b57b-526539ccdba2
TCGA-GBM	TCGA-32-4213-01A	Tumor	b004e38d-f48c-49f9-a027-4a702fbaf107
TCGA-GBM	TCGA-19-2625-01A	Tumor	95e82e2f-d87e-4785-9e8e-c6b41d8b73f0
TCGA-GBM	TCGA-06-0675-11A	Normal	9b571cc1-447f-4fa1-b797-bf95c1ee2372
TCGA-GBM	TCGA-06-0681-11A	Normal	026527d4-007c-4c4e-9bd5-855c44bbe7b0
TCGA-GBM	TCGA-26-5136-01B	Tumor	04be0c6b-c75f-40cf-82c0-c14af4f9e456
TCGA-GBM	TCGA-06-2570-01A	Tumor	bdba7172-5280-41f6-8082-dbf8983c4089
TCGA-GBM	TCGA-15-1444-01A	Tumor	effc6b43-7853-4b65-b6a1-265ea15f2846
TCGA-GBM	TCGA-06-0211-01A	Tumor	c904090b-149e-4146-9769-f0d2ff965da9
TCGA-GBM	TCGA-06-2563-01A	Tumor	742b41f9-faca-4bad-a2a5-5d40b8bf06ce
TCGA-GBM	TCGA-06-0645-01A	Tumor	c3d1dc80-eea8-43f8-8436-5bd0ee6a3b07
TCGA-GBM	TCGA-06-0678-11A	Normal	8880fb74-0849-422e-bfd5-4a38c5a0a906
TCGA-GBM	TCGA-06-1804-01A	Tumor	eb80bbc4-7186-4872-86df-90629a53ecb1
TCGA-GBM	TCGA-06-0190-01A	Tumor	982e2940-10b0-4383-bde8-f7ccb9920fc4
TCGA-GBM	TCGA-06-0210-01A	Tumor	90629e6e-2e1e-4f31-884a-6bca2c24ce11
TCGA-GBM	TCGA-06-0882-01A	Tumor	7376f374-1439-405e-87d3-c45cfac92ed5
TCGA-GBM	TCGA-06-0744-01A	Tumor	e52fefef-84a4-4b1d-91e2-9e9735e19e1f
TCGA-GBM	TCGA-12-1597-01B	Tumor	c6045584-592e-412f-974a-c8ae2d4bb817
TCGA-GBM	TCGA-32-1970-01A	Tumor	44067d6e-af12-4abf-8795-cb6749b1cc7a
TCGA-GBM	TCGA-28-2499-01A	Tumor	270906c7-7a4c-4684-8c48-c4e337f5d9fc
TCGA-GBM	TCGA-06-AABW-11A	Normal	63a30223-e2e9-45bc-bc42-c00f9202b493
TCGA-GBM	TCGA-27-2524-01A	Tumor	02654be6-2049-4000-a0ac-c26f7ba6f0c9
TCGA-GBM	TCGA-06-5414-01A	Tumor	8b3a593a-2359-476a-a501-e1baaa4873bb
TCGA-GBM	TCGA-12-5295-01A	Tumor	a75c6098-ff48-43c2-b56d-6fa448ddebfb
TCGA-GBM	TCGA-02-2483-01A	Tumor	19f1375f-51f2-4f4c-88fe-6fc84c37e5b6
TCGA-GBM	TCGA-14-1825-01A	Tumor	1ed5fd9b-a682-4e81-bda1-7ed085f4355d
TCGA-GBM	TCGA-27-2521-01A	Tumor	c42fa4ad-4f31-4dfd-b499-599896a57517
TCGA-GBM	TCGA-28-5208-01A	Tumor	adce8dd4-e2df-43ed-9821-3781ddb7a82f
TCGA-GBM	TCGA-32-2615-01A	Tumor	1ede5c59-96ae-486b-ae1f-5f7c7909cf6a
TCGA-GBM	TCGA-06-0187-01A	Tumor	f2d5627a-f891-453b-8bdb-38d732c59342
TCGA-GBM	TCGA-06-0174-01A	Tumor	c1fdf09e-3960-4aaa-8c49-fe64c6c5bcea
TCGA-GBM	TCGA-06-2567-01A	Tumor	a5461e7c-620e-49c0-b4bf-f9774b9393af
TCGA-GBM	TCGA-06-0238-01A	Tumor	01521666-f595-4074-aea1-f7ab78db062b
TCGA-GBM	TCGA-76-4928-01B	Tumor	cbc1bf1c-8ddd-424a-b95a-5ca231d94e63
TCGA-GBM	TCGA-19-2620-01A	Tumor	37897de3-c6da-4f80-bf9d-a36ee2cf5e15
TCGA-GBM	TCGA-26-5139-01A	Tumor	8917fe2a-c1bb-440c-8ea5-655504006a63
TCGA-GBM	TCGA-06-0680-11A	Normal	d50c9013-3aee-4b1f-be93-225e568da7c3
TCGA-GBM	TCGA-06-5413-01A	Tumor	4befb3ab-b606-4035-a0b0-639032962767
TCGA-GBM	TCGA-28-2509-01A	Tumor	b888bce3-6b9e-494b-b1e8-13714c8b5267
TCGA-GBM	TCGA-28-5213-01A	Tumor	84aa891c-c73e-48eb-bdbe-5774c2dc3c58
TCGA-GBM	TCGA-06-0649-01B	Tumor	6bdfd682-d5da-403d-b901-9d6fec360d3a
TCGA-GBM	TCGA-14-0790-01B	Tumor	e13a37ca-e668-4315-b156-f4eeb5814c05
TCGA-GBM	TCGA-06-5858-01A	Tumor	d90b02f3-4c68-40ec-9704-1d1d6706e402

TCGA-GBM	TCGA-26-1442-01A	Tumor	3462c776-160b-4ffc-9be8-69b08abf9780
TCGA-GBM	TCGA-12-5299-01A	Tumor	f6a99d72-7ef2-4c9c-880c-a26bd5fef9c7
TCGA-GBM	TCGA-06-2569-01A	Tumor	bd7b2c7c-3a38-4a2b-b6eb-5f67572db883
TCGA-GBM	TCGA-06-0132-01A	Tumor	50a6757f-496c-4d2d-9463-f948c09c28f6
TCGA-GBM	TCGA-06-0168-01A	Tumor	ceb8fbdf-dd36-4273-b17c-0ee2eca493c5
TCGA-GBM	TCGA-02-2485-01A	Tumor	1a860864-2ba1-46ad-ad36-018a7b0ec08e
TCGA-GBM	TCGA-14-0789-01A	Tumor	fffc1088-c5a6-46a0-b050-860184f6ded2
TCGA-GBM	TCGA-27-2523-01A	Tumor	4ea9f653-d97f-4e04-ad32-620017803919
TCGA-GBM	TCGA-19-1390-01A	Tumor	cbdf8c09-e3f4-4b3e-bdaa-f4a3a4e40068
TCGA-GBM	TCGA-06-0157-01A	Tumor	2c15cc20-2347-4bfc-826d-a062f6d74b46
TCGA-GBM	TCGA-27-1831-01A	Tumor	e2a01ce0-a2fb-435a-9b05-7758e14d4157
TCGA-GBM	TCGA-16-1045-01B	Tumor	e87cfbaa-791e-4a6b-8ec8-28df636e7cdc
TCGA-GBM	TCGA-19-4065-01A	Tumor	bc8b9c8b-d288-436e-9e33-8c31a9575ce7
TCGA-GBM	TCGA-41-2572-01A	Tumor	21a4485e-a6e0-4129-8148-3d092ebbdd90

Supplementary Table 4. Primers used for RT-qPCR

Gene	Primer Sequences	Product Size	Source
GADPH	Forward 5'- GAGTCAACGGATTTGGTCGT -3'<br>Reverse 5'- TTGATTTTGGAGGGATCTCG -3'	238 bp	Wang et al. [1]<br>Wang et al. [2]<br>Kikuno et al. [3]
MMP9	Forward 5'- TTGACAGCGACAAGAAGTGG -3'<br>Reverse 5'- GCCATTCACGTCGTCCTTAT -3'	179 bp	Uehara et al. [4]<br>Kikuno et al. [3]
TMEM92	Forward 5′- AGCCAAATGTGGTCTCATCC -3′,<br>Reverse 5′- CCAGGAAGATGATGACGAAGA -3′	119 bp	da Cunha et al. [5]
C1orf226	Forward 5’- GCAGGAGGTGACACTCTCAA -3’<br>Reverse 5’- CGTGGTCAACTGTCCGAGAA -3’	89 bp	This study
CD163	Forward 5’- CTGGCGTGACATGTTCTGAT -3’<br>Reverse 5’- CAGTCTCTGAATCTCCACCTCAAC -3’	98 bp	da Cunha et al. [5]<br>and this study
AK5	Forward 5′- GCTGCTCCATTGGTTAAATACTTCC -3′<br>Reverse 5′- GTTGTCAACTGCCATGCTGATG -3′	108 bp	Saito et al. [6]
CCR7	Forward 5’-CCCTCCCTCCATCGTTTTC-3’<br>Reverse 5’- CACCACACTCTCCCCTGTTG -3’	134 bp	This study
CD200	Forward 5'- CCTAAGAATCAGGTGGGGAAGGA -3'<br>Reverse 5'- GACGAGAAGAATTACCAGGGAAACA -3'	137 bp	Pontikoglou et al. [7]
MICU3	Forward 5'- ACCATCAGTGAAGAAGATTTTGCTC -3'<br>Reverse 5'- TGTGATGCCCTTTTCTTCAGGT -3'	114 bp	This study
18S rRNA	Forward 5'- GGCCCTGTAATTGGAATGAGTC -3'<br>Reverse 5'- CCCAAGATCCAACTACGAGCTTT -3	147 bp	Chen et al.[8]<br>and this study
LINC00482	Forward 5'- CTCTGTGGGAGCCTAGATGG -3'<br>Reverse 5'- CCATAGCCCTTCTTAACGCC -3'	135 bp	This study
U6	Forward: QIAGEN PCR Control Hs_RNU6-2_1 Primer<br>Reverse: QIAGEN Universal Primer	N/A	This study
hsa-miR-3918	Forward 5'- CACGAAACAGGGCCGCAG -3'<br>Reverse: QIAGEN Universal Primer	N/A	This study
hsa-miR-760	Forward 5’- CCCGGCTCTGGGTCTGTG -3'<br>Reverse: QIAGEN Universal Primer	N/A	This study

N/A, not applicable.

Supplementary References

1. Wang, F. *et al.* Identification of a panel of genes as a prognostic biomarker for glioblastoma. *EBioMedicine* **37**, 68-77, doi:10.1016/j.ebiom.2018.10.024 (2018).
2. Wang, F. *et al.* Nucleolin Is a Functional Binding Protein for Salinomycin in Neuroblastoma Stem Cells. *J Am Chem Soc* **141**, 3613-3622, doi:10.1021/jacs.8b12872 (2019).
3. Kikuno, N. *et al.* Knockdown of astrocyte-elevated gene-1 inhibits prostate cancer progression through upregulation of FOXO3a activity. *Oncogene* **26**, 7647-7655, doi:10.1038/sj.onc.1210572 (2007).
4. Uehara, O. *et al.* Upregulated expression of MMP-9 in gingival epithelial cells induced by prolonged stimulation with arecoline. *Oncol. Lett.* **14**, 1186-1192, doi:10.3892/ol.2017.6194 (2017).
5. da Cunha, J. P. *et al.* Bioinformatics construction of the human cell surfaceome. *Proc. Natl. Acad. Sci. U. S. A.* **106**, 16752-16757, doi:10.1073/pnas.0907939106 (2009).
6. Saito, Y. *et al.* Identification of therapeutic targets for quiescent, chemotherapy-resistant human leukemia stem cells. *Sci. Transl. Med.* **2**, 17ra19, doi:10.1126/scitranslmed.3000349 (2010).
7. Pontikoglou, C. *et al.* CD200 expression in human cultured bone marrow mesenchymal stem cells is induced by pro-osteogenic and pro-inflammatory cues. *J. Cell. Mol. Med.* **20**, 655-665, doi:10.1111/jcmm.12752 (2016).
8. Chen, C. *et al.* PDE8A genetic variation, polycystic ovary syndrome and androgen levels in women. *Mol. Hum. Reprod.* **15**, 459-469, doi:10.1093/molehr/gap035 (2009).
