# Supplementary material for: Transcriptomic analyses of patient peripheral blood with hemoglobin depletion reveal glioblastoma biomarkers
Source: NPJ Genom Med. 2023 Jan 25;8:2. doi: 10.1038/s41525-022-00348-3 (PMC9877004; doi:10.1038/s41525-022-00348-3)
Supplement: Supplementary file 2 — Reporting Summary [file 41525_2022_348_MOESM2_ESM.pdf]

nature portfolio

Corresponding author(s): Erxi Wu

Last updated by author(s): Nov 5, 2022

Reporting Summary

Nature Portfolio wishes to improve the reproducibility of the work that we publish. This form provides structure for consistency and transparency in reporting. For further information on Nature Portfolio policies, see our Editorial Policies and the Editorial Policy Checklist.

Statistics

For all statistical analyses, confirm that the following items are present in the figure legend, table legend, main text, or Methods section.

n/a	Confirmed	
☐	☒	The exact sample size (*n*) for each experimental group/condition, given as a discrete number and unit of measurement
☐	☒	A statement on whether measurements were taken from distinct samples or whether the same sample was measured repeatedly
☐	☒	The statistical test(s) used AND whether they are one- or two-sided<br>*Only common tests should be described solely by name; describe more complex techniques in the Methods section.*
☐	☒	A description of all covariates tested
☐	☒	A description of any assumptions or corrections, such as tests of normality and adjustment for multiple comparisons
☐	☒	A full description of the statistical parameters including central tendency (e.g. means) or other basic estimates (e.g. regression coefficient) AND variation (e.g. standard deviation) or associated estimates of uncertainty (e.g. confidence intervals)
☐	☒	For null hypothesis testing, the test statistic (e.g. *F*, *t*, *r*) with confidence intervals, effect sizes, degrees of freedom and *P* value noted<br>*Give P values as exact values whenever suitable.*
☒	☐	For Bayesian analysis, information on the choice of priors and Markov chain Monte Carlo settings
☒	☐	For hierarchical and complex designs, identification of the appropriate level for tests and full reporting of outcomes
☐	☒	Estimates of effect sizes (e.g. Cohen's *d*, Pearson's *r*), indicating how they were calculated

Our web collection on statistics for biologists contains articles on many of the points above.

Software and code

Policy information about availability of computer code

Data collection	RNA-seq was performed using Illumina HiSeq 2000 and BGISEQ-500 at Beth Israel Deaconess Medical Center (BIDMC) Genomics Proteomics Core at Harvard Medical School. TCGA data were downloaded from TCGA database. Datasets from the R2 Genomics Analysis and Visualization Platform were collected, analyzed and visualized on their website.
Data analysis	Data obtained from our study were processed using CRAN R (version 4.1.0, 4.1.2 and 4.2.1), and R studio (version v1.3.1073, v1.4.1717, v2022.02.3+492 and v2022.07.1+554). Sequencing data processing was performed using FASTQC, HISAT2, the Ensembl human genome GRCh38 reference assembly, miRBase v21. Pathway analysis was performed via the Reactome Pathway Database online analysis tools. Key open sources for data analysis are DESeq2, limma, edgeR, multiMiR, DCModule Generation v2, ggpubr, rstatix, ggplot2, ComplexHeatmap, and igraph.

For manuscripts utilizing custom algorithms or software that are central to the research but not yet described in published literature, software must be made available to editors and reviewers. We strongly encourage code deposition in a community repository (e.g. GitHub). See the Nature Portfolio guidelines for submitting code & software for further information.

Data

Policy information about availability of data

All manuscripts must include a data availability statement. This statement should provide the following information, where applicable:

- Accession codes, unique identifiers, or web links for publicly available datasets
- A description of any restrictions on data availability
- For clinical datasets or third party data, please ensure that the statement adheres to our policy

Data generated from this study have been included in the manuscript files. Publicly available databases (https://www.cancer.gov/tcga; http://r2.amc.nl; https://reactome.org/) used in this study have been cited, and detailed information has been provided in this manuscript. The availability of blood sequencing data should be requested and addressed to Dr. Erxi Wu, Erxi.Wu@BSWHealth.org. The sequencing raw data and processed count tables of this study were deposited into the NCBI Sequence Read Archive (BioProject ID: PRJNA878767) and Github (https://github.com/ddqq666/gProj), respectively.

Human research participants

Policy information about studies involving human research participants and Sex and Gender in Research.

Reporting on sex and gender	Glioblastomas are diagnosed in both males and females. Therefore, no exclusion of subjects was based on sex in this study. Blood RNA-seq data were analyzed with adjustment for sex. Analysis of external datasets (e.g. TCGA data) was not sex-controlled since sex data are not reported for samples in the normal control group.
Population characteristics	Adult patients diagnosed with glioblastoma and adult individuals with no cancer diagnosis as controls were included in this study. Children were not included in this study. No exclusion was based on race and ethnicity in this study.
Recruitment	The inclusion criteria were as follows: i) adult patients diagnosed with glioblastoma (no matter de novo or secondary) are includable, ii) patients with noncancer comorbid conditions such as hypertension, headache, obesity, and kidney diseases are acceptable, and iii) adults without cancer diagnosed are acceptable as noncancer controls. The exclusion criteria were i) patients with other types of cancer or brain metastasis and ii) patients with infectious diseases. There was no exclusion defined based on the sex, race or ethnicity of the subjects.
Ethics oversight	The Institutional Review Board of Baylor Scott & White Health

Note that full information on the approval of the study protocol must also be provided in the manuscript.

Field-specific reporting

Please select the one below that is the best fit for your research. If you are not sure, read the appropriate sections before making your selection.

☒ Life sciences ☐ Behavioural & social sciences ☐ Ecological, evolutionary & environmental sciences

For a reference copy of the document with all sections, see nature.com/documents/nr-reporting-summary-flat.pdf

Life sciences study design

All studies must disclose on these points even when the disclosure is negative.

Sample size	Samples were used to analyze differential gene expression. The sample size was sufficient to achieve significance with an adjusted p-value (false discovery rate) of less than 5%. Data were also tested in external datasets to reduce false positives.
Data exclusions	Sequencing data that did not pass quality control were excluded.
Replication	Transcriptomic data from this study or external datasets are one value per gene per sample due to availability. PCR experiments were performed three times per gene per sample and are presented as the mean with standard deviation in the supplementary material.
Randomization	This is not a clinical trial study. Subjects in this study were randomly included if they met the recruitment criteria and agreed to participate.
Blinding	Subjects were deidentified and designated with numbers. The investigator and contributors in this study did not have any identity information about the included subjects.

Reporting for specific materials, systems and methods

We require information from authors about some types of materials, experimental systems and methods used in many studies. Here, indicate whether each material, system or method listed is relevant to your study. If you are not sure if a list item applies to your research, read the appropriate section before selecting a response.

Materials & experimental systems

n/a	Involved in the study
☒	☐ Antibodies
☒	☐ Eukaryotic cell lines
☒	☐ Palaeontology and archaeology
☒	☐ Animals and other organisms
☒	☐ Clinical data
☒	☐ Dual use research of concern

Methods

n/a	Involved in the study
☒	☐ ChIP-seq
☒	☐ Flow cytometry
☒	☐ MRI-based neuroimaging
